# The Population Structure of *Borrelia lusitaniae* Is Reflected by a Population Division of Its *Ixodes* Vector

**DOI:** 10.3390/microorganisms9050933

**Published:** 2021-04-27

**Authors:** Ana Cláudia Norte, Pierre H. Boyer, Santiago Castillo-Ramirez, Michal Chvostáč, Mohand O. Brahami, Robert E. Rollins, Tom Woudenberg, Yuliya M. Didyk, Marketa Derdakova, Maria Sofia Núncio, Isabel Lopes de Carvalho, Gabriele Margos, Volker Fingerle

**Affiliations:** 1 MARE-Marine and Environmental Sciences Centre, Department of Life Sciences, University of Coimbra, 3000-456 Coimbra, Portugal; acgnorte@ci.uc.pt; 2 Centre for Vector and Infectious Diseases Research, National Institute of Health Doutor Ricardo Jorge, Águas de Moura, 2965-575 Setúbal, Portugal; sofia.nuncio@insa.min-saude.pt (M.S.N.); isabel.carvalho@insa.min-saude.pt (I.L.d.C.); 3 CHRU Strasbourg, UR7290 Lyme Borreliosis Group, ITI InnoVec, Fédération de Médecine Translationnelle de Strasbourg, Institut de Bactériologie, University of Strasbourg, 3 rue Koeberlé, 67000 Strasbourg, France; pierreboyer@unistra.fr; 4 Programa de Genómica Evolutiva, Centro de Ciencias Genómicas, Universidad Nacional Autónoma de México, Apartado Postal 565-A, Cuernavaca, CP 62210, Mexico; iago@ccg.unam.mx; 5 Institute of Zoology, Slovak Academy of Sciences, 84506 Bratislava, Slovakia; michalchvostac@gmail.com (M.C.); yu.m.didyk@gmail.com (Y.M.D.); marketa.derdakova@gmail.com (M.D.); 6 Laboratory of Ecology and Biology of Terrestrial Ecosystems, Faculty Biological and Agronomic Sciences, University Mouloud Mammeri, 15000 Tizi-Ouzou, Algeria; brahamimohand@yahoo.fr; 7 Division of Evolutionary Biology, LMU Munich, Faculty of Biology, Grosshaderner Strasse 2, 82152 Planegg-Martinsried, Germany; rollins@biologie.uni-muenchen.de; 8 National Reference Center for Borrelia, Bavarian Health and Food Safety Authority, 85764 Oberschleissheim, Germany; tom.woudenberg@gmail.com (T.W.); volker.fingerle@lgl.bayern.de (V.F.); 9 Department of Acarology, I. I. Schmalhausen Institute of Zoology, National Academy of Sciences of Ukraine, B. Khmelnytskogo 15, 01030 Kyiv, Ukraine; 10 Environmental Health Institute, Medicine Faculty, University of Lisbon, 1649-026 Lisbon, Portugal

**Keywords:** population structure, vector association, *Borrelia lusitaniae*, Ixodes, MLST

## Abstract

Populations of vector-borne pathogens are shaped by the distribution and movement of vector and reservoir hosts. To study what impact host and vector association have on tick-borne pathogens, we investigated the population structure of *Borrelia lusitaniae* using multilocus sequence typing (MLST). Novel sequences were acquired from questing ticks collected in multiple North African and European locations and were supplemented by publicly available sequences at the Borrelia Pubmlst database (accessed on 11 February 2020). Population structure of *B. lusitaniae* was inferred using clustering and network analyses. Maximum likelihood phylogenies for two molecular tick markers (the mitochondrial 16S rRNA locus and a nuclear locus, Tick-receptor of outer surface protein A, *trospA*) were used to confirm the morphological species identification of collected ticks. Our results confirmed that *B. lusitaniae* does indeed form two distinguishable populations: one containing mostly European samples and the other mostly Portuguese and North African samples. Of interest, Portuguese samples clustered largely based on being from north (European) or south (North African) of the river Targus. As two different *Ixodes* species (i.e., *I. ricinus* and *I. inopinatus*) may vector *Borrelia* in these regions, reference samples were included for *I. inopinatus* but did not form monophyletic clades in either tree, suggesting some misidentification. Even so, the *trospA* phylogeny showed a monophyletic clade containing tick samples from Northern Africa and Portugal south of the river Tagus suggesting a population division in *Ixodes* on this locus. The pattern mirrored the clustering of *B. lusitaniae* samples, suggesting a potential co-evolution between tick and *Borrelia* populations that deserve further investigation.

## 1. Introduction

Populations of vector-borne infectious disease agents are shaped by migration of, and selection pressure exerted by, their hosts and vectors [1,2,3,4] and these processes leave genetic signatures in the genomes of the organisms. Investigation of the geographical distribution of genetic lineages and their diversity permit inference of evolutionary processes such as selection or migration that have shaped populations [4]. Ticks range among the most important vectors for microbial pathogens [5] but many pathogens they transmit are difficult to study [6]. Disease agents that have been associated with *Ixodes* ticks include *Borrelia* spp., *Babesia* spp., *Anaplasma* spp., *Rickettsia* spp., and viruses such as tick-borne encephalitis virus or Powassan virus with species varying in their geographical distribution and pathogenic potential [5], [7]. The abundance of ticks, tick-borne disease agents and resulting host-parasite interactions have been reported to be influenced by environmental changes, among them changes in land use, increases in temperature due to climate change and others [3,8,9]. In this study, as a proxy for tick-transmitted pathogens, we investigated a population of the most frequently found microbes transmitted by ticks in the temperate northern hemisphere, i.e., microbes that belong to the *Borrelia burgdorferi* sensu lato species complex.

The *Borrelia burgdorferi* sensu lato species complex comprises more than 20 spirochetal bacterial species that use ticks of the genus *Ixodes* as vector and small to medium sized vertebrate species as reservoir hosts. *Borrelia* species vary considerably in the scale of their host and vector specificity [1,6,10,11], and this is reflected in the geographic distribution of their lineages [11,12,13]. Furthermore, host- and vector associations have been identified as drivers for diversification and speciation in *Borrelia* [6,11,14,15]. For example, *B. burgdorferi* sensu stricto (s.s.) Johnson et al. 1984, the main cause of human Lyme borreliosis in North America, is considered a generalist species because it can have different groups of vertebrate species such as rodents or birds as reservoir hosts and several *Ixodes* species as vectors [16,17]. The distribution range in North America includes the Northeast and Midwest of the USA [18] ranging now into Canada [19,20,21] and Western coastal regions [22,23]. Phylogenies based on multilocus sequence typing (MLST) or genomic SNPs have revealed a complex population structure, but strains do not cluster according to geography [24,25,26,27]. Species using only avian reservoirs, such as *Borrelia garinii* Baranton et al. 1982 or *Borrelia valaisiana* Wang et al. 1997, showed very little population structure likely owing to the migration and dispersal pattern of their avian reservoir hosts [12,13,28,29,30,31,32]. In comparison, species that use hosts with smaller migration ranges such as rodents or lizards show more pronounced population structure, supposedly reflecting the migration range of their hosts [12,33].

*Borrelia lusitaniae* Le Fleche et al. 1997 was isolated for the first time in Portugal [34] and described as a species in 1997 [35]. The species has been associated with lizards as reservoir hosts [36,37,38] and *Ixodes ricinus* Linnaeus, 1758 as its main vector [39,40,41]. Several species of the family Lacertidae, e.g., *Psammodromus algirus* Linnaeus, 1758 [36,38,42], *Podarcis* spp. Wagler, 1830 [37,38,43,44], *Teira dugesii* Milne-Edwards 1829 [45] as well as *Lacerta* spp. Linnaeus, 1758 [43,46] were incriminated as potential reservoir hosts for *B. lusitaniae*. Some studies reported isolation of *B. lusitaniae* from, or detection of *B. lusitaniae* by PCR in, larvae feeding on the rodent *Apodemus sylvaticus* Linnaeus, 1758 [38,47]. Although this is not proof of reservoir competence, it cannot be immediately discounted that hosts other than lizards may serve as reservoirs for *B. lusitaniae*; further studies are needed to clarify this point. Initial reports of *B. lusitaniae* came from around the Mediterranean basin (Tunisia, Morocco, Portugal, Italy) [34,41,48,49,50] but now it has also been reported in countries such as Slovakia, Poland, and Latvia [46,51,52,53]. Remarkably high *Borrelia* infection rates of adult ticks (at the time identified as *I. ricinus*) (75%, 41 out of 55) were found in Portugal near Grândola of which a majority were identified as *B. lusitaniae* [54] suggesting favorable conditions in terms of reservoir hosts and vector ticks for this *Borrelia* species. First studies showed that *B. lusitaniae* induced disease in C3H/HeN mice, causing both nodular interstitial cystitis and vasculitis of the great vessels of the heart [55]. From an epidemiological perspective, further investigations of *B. lusitaniae* populations will be important as to date two isolates have been obtained from human clinical cases [56,57,58].

The population structure of *B. lusitaniae* was analyzed based on single genes (outer surface protein A; *ospA*) [59] and multilocus sequence typing (MLST) [33]. The study on *ospA* included samples from Italy, Germany, Morocco and Portugal. Phylogenetic analyses demonstrated that Portuguese tick isolates (PoTiB1, PoTiB2 and PoTiB3, all isolated from ticks from around Águas de Moura, south of the river Tagus) formed a clade together with North African isolates while isolates from Germany, Italy and a Portuguese human isolate (from Lisbon region) formed a second clade [59]. The MLST study included samples from Portugal collected in Mafra (north of the river Tagus) and Grândola (south of the river Tagus). Phylogenetic analyses of MLST sequences showed that isolates from north of the Tagus river mostly clustered together while samples from Grândola formed a second cluster suggesting two populations in Portugal–one from the north of the Tagus river, the other one from south of the Tagus river [33]. To confirm the population structure of *B. lusitaniae*, we analyzed, using MLST, samples from various European countries (Austria, Croatia, Latvia, Serbia, Slovakia, Ukraine and Portugal) and from North Africa (Algeria). The Portuguese samples included specimens from north (Mafra and Coimbra) and south (Santiago do Cacém) of the river Tagus and from Madeira island. In addition, we tested the hypothesis that the population structure of *B. lusitaniae* is shaped by association with different vector species. In 2014, a new *Ixodes* species was described and named *Ixodes inopinatus* Estrada-Peña, Nava et Petney 2014 [60]. The authors describe that *I. inopinatus* can be found on lizards and suggest that it replaces *I. ricinus* in dry Mediterranean regions of Algeria, Morocco, Portugal, Spain and Tunisia. A previous study on the population structure of *I. ricinus* had shown that there was a divergent clade in North Africa [61]. The latter study used concatenated sequences of four mitochondrial and nuclear genes including the 16S rRNA locus and Tick-receptor of outer surface protein A (*trospA*). In the present study, we used these two loci to establish whether there are different populations of *Ixodes* in Portugal and whether the ticks collected in Algeria may be identified as *I. inopinatus*.

## 2. Materials and Methods

### 2.1. Tick Collection and Processing

Questing ticks were collected by standardized flagging method [62]. Ticks from Algeria were removed from cattle, one specimen was collected in Portugal feeding on a *P. algirus* (PoTiB11/T3087), three specimens were collected in 2018 in Germany from great tits *Parus major* Linnaeus, 1758 (11-E12, 7-F5, 8-C12) [63], and three specimens feeding on humans (13310PT18, 13360PT18 and 14401PT18). Collected ticks were morphologically determined into stages and species [60,64] and stored in 70% ethanol until further analyses, except for one sample that was inoculated in BSK medium (see Table S1).

In Slovakia, ticks were collected in years 2013–2017 in an ecotone between the forest and a small mountain stream on Martinské hole mountains (49.085464, 18.863042; 630–660 m ASL). The vegetation was composed mainly of beech, spruce, hazel and elderberry with herbal undergrowth.

In Croatia, ticks were collected in 2011 in Grabovac (44.971317, 15.640267; 420 m ASL). The study area consisted of hornbeam forest, shrubs and pastures.

In Ukraine, ticks were collected during 2015–2016 in urban green areas Kyiv city: (50.446639, 30.570000; 179 m ASL). The vegetation cover at the sampling sites consisted of deciduous forests, shrubs and inhomogeneous meadows.

In Algeria, ticks were collected during 2015–2016 from cattle, in the mountainous Akfadou forest. Three collection sites were investigated during the collection campaign, which are places where cattle rest and can drink: site A (36.6289361, 4.57211111; 1567 m ASL); site B (36.690625, 4.57448611; 1196 m ASL) and site C (36.6951028, 4.55724694; 1201 m ASL).

Questing ticks from Portugal were collected in Tapada de Mafra in 2003 and 2013, and in 2009 in the Madeira Archipelago, Parque Natural Peneda-Gerês, and in Santiago do Cacém. Details on vegetation and fauna are given in Supplementary Material.

**Figure 1:**
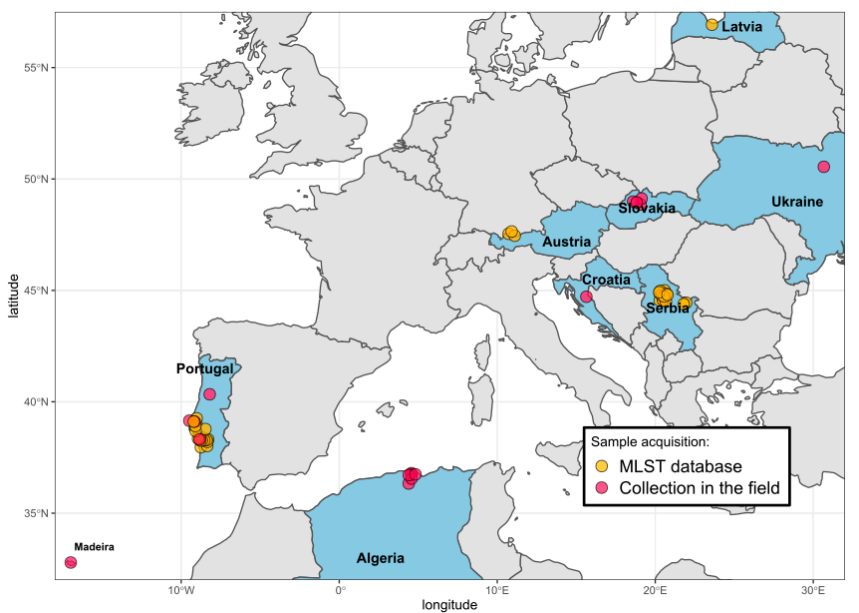
Map of countries from which ticks and *B. lusitaniae* were included in this study. Samples from Austria, Latvia, Serbia and some samples from Portugal were retrieved from the MLST website. The locations of sample collection were slightly shifted for visualization purposes. The map was generated in R. For details, see Table 1 and Table 2, Tables S1 and S2.

Countries where ticks were collected and *Borrelia* samples characterized are shown in Figure 1. Details on geographic origin, species and number of ticks included in the current analysis are given in Table 1 and Table S1.

### 2.2. DNA Extraction

For DNA isolation from ticks, different methods were used. In Slovakia and Ukraine, alkaline-hydrolysis was used as described [65] on individual ticks. In Portugal, a DNAeasy kit (Qiagen, Hilden, Germany) was used. Ticks sampled in Algeria were individually extracted using the MagNA Pure 96 system using the MagNA Pure96 DNA and Viral NA Small Volume Kit (Roche Diagnostics, Meylan, France) according to the manufacturer’s instruction and for ticks from Germany the Qiagen BioSprint 96 DNA Blood kit (Qiagen) was used. DNA samples were stored frozen (at −20 °C and −80 °C, respectively), until further processing. In samples from Croatia, Slovakia, and Ukraine the presence of tick DNA was confirmed by amplification of a 620 bp fragment of the tick mitochondrial gene cytochrome B (Black & Roehrdanz 1998). Only PCR positive samples were further analyzed.

### 2.3. PCR Borrelia Genes

*Borrelia burgdorferi* s.l. was detected through PCR amplification of a 222–255 bp fragment of the *rrfA*-*rrlB* intergenic spacer (IGS) in samples from Croatia, Slovakia, and Ukraine [66] and Portugal [67]. In Portuguese and Algerian samples *B. burgdorferi* s.l. was confirmed through amplification of the flagellin B (*flaB*) gene [68,69] (see Table S2).

MLST on *Borrelia* positive samples was done primarily using a nested or semi-nested PCR (HotStar Taq Master Mix Kit or HotStarTaq Plus DNA Polymerase kit, Qiagen, Hilden, Germany), with primers and PCR conditions as previously described [70,71] [see also https://pubmlst.org/borrelia/ (11 February 2020)]. As the *uvrA* gene did not amplify in all cases, fragments of the seven housekeeping genes *clpA*, *clpX*, *nifS*, *pepX*, *pyrG*, *recG*, and *rplB* were amplified and sequenced. As positive control DNA of *B. japonica* or *B. turdi* was used while double distilled water was used as negative control. Table 2 and Table S2 give an overview of *B. lusitaniae* positive samples that were typed by MLST and for which at least seven MLST housekeeping genes gave good sequences without ambiguities. MLST sequences were submitted and are available via pubmlst.org/borrelia/ (11 May 2020) at the University of Oxford.

Purification of PCR products was done using a NucleoSpin Gel and PCR Clean-up according to the manufacturer’s recommendation (MACHEREY-NAGEL, Düren, Germany). For Slovakian and Algerian samples Sanger sequencing of all the genes was performed by Eurofins Genomics (Eurofins Genomics Germany GmbH, Ebersberg, Germany and Basel, Switzerland).

### 2.4. PCR on Tick Genes

To confirm the morphological identification of ticks and to differentiate *I. ricinus* and *I. inopinatus*, two previously used loci, the mitochondrial 16S rRNA locus and the nuclear gene *trospA* were amplified [61,72] and sequenced. We included tick samples that were characterized for *Borrelia* or originated from the same regions as the *Borrelia* positive tick samples, but they were not necessarily identical. Three morphologically identified samples that matched the 16S rRNA of *I. inopinatus* were available, designated as such, and were included as controls (for details see Table S1). In tick samples from Algeria, Slovakia and Portugal, the 16S rRNA gene was amplified according to [72] and the *trospA* locus was amplified using primers and conditions with slight modification according to [61]. The implemented modification was a touch-down protocol for the initial six rounds of amplification to minimize non-specific background; the annealing temperatures started at 61 °C and decreased 1 °C per cycle until a temperature of 56 °C was reached. Further amplification rounds were performed at 56 °C. Further details on PCR conditions are given in supplementary information.

### 2.5. Bioinformatic Analysis Recombination and Network Analysis on MLST Genes of Borrelia Samples

Recombination signals were analyzed in the MLST sequence alignment and in both *trospA* and 16S rRNA sequences employing the PhiTest implemented in SplitTree4 [73]. Because the MLST alignment had recombination signals NeighborNet was run instead of a regular phylogeny; we used SplitTree4 to construct the net and selected the uncorrected P distance and equal angle display.

To identify the potential recombination events in the MLST alignment we used Gubbins with default parameters [74]. We also conduct individual recombination analysis via PhiPack [75], following the method described for *A. baumannii* [76], on each the fragments of the seven housekeeping genes employed for the MLST. Of note, out of the seven housekeeping genes in *clpA* we found signals of recombination (*p*-value = 3.66 × 10^−2^).

### 2.6. Hierarchical Population Structure Analysis (HPSA) of Borrelia

For a hierarchical population structure analysis using hierBAPS [77], we used the alignment of concatenated MLST sequences. The analysis was run at four levels of clustering and the number of initial clusters was set to 20. We also computed the coefficient of differentiation via MEGA X [78] for the two major groups found at the first level of HPSA clustering.

### 2.7. Hierarchical Clustering of MLST Genes of Borrelia Samples

To assess the hypothesis of having two genetic clades in Europe, we applied a hierarchical clustering algorithm [79]. This algorithm aggregated the isolates towards one group using the allelic profile data on the seven genes (*clpA*, *clpX*, *nifS*, *pepX*, *pyrG*, *recG* and *rplB*). The hierarchical clustering algorithm treats every isolate as its own cluster and will then look for the closest related isolate based on a similarity measure (allelic profile) and fuses these into a cluster. This step is repeated until all isolates belong to a cluster which can be visualized in a dendrogram. The similarity measure was Gower’s similarity coefficient [80]. This coefficient can range from 0 to 1, where a score of 1 indicates complete similarity. Identical isolates fuse directly in the dendrogram, whereas dissimilar isolates only fuse at a more upward branch. R (version 4.0.2) [81] was used to map the data with package “rnaturalearth” and “ggplot2”. The dendrogram was build using the R package “dendextend” [82].

### 2.8. GoeBURST Analysis of Borrelia

We used PHYLOVIZ 1.1 [83] to conduct goeBURST analyses using the triple locus variant setting [84] using ST numbers, allelic profiles and meta information. Previous data available for *B. lusitaniae* isolates were downloaded from the *Borrelia* MLST database [www.pubmlst.org/borrelia/ (11 February 2020)] and included in our network and goeBURST analyses.

### 2.9. Maximum Likelihood Phylogenies of Tick Samples

As 16S rRNA and *trospA* did not show signals of recombination regular phylogenies were constructed. For both loci, the best model for the phylogeny was selected via jModelTest [85]. For each locus, a maximum likelihood (ML) phylogeny was run using PhyML [86] setting the best model, which was HK+I for 16S and K80+I for *trospA*.

## 3. Results

### 3.1. Identification of Borrelia lusitaniae in Ticks

Ticks collected in Europe (Croatia, Portugal, Slovakia, Ukraine,) and North Africa (Algeria) that tested positive for *B. burgdorferi* in screening PCRs were further analyzed by MLST to identify *B. lusitaniae*. Details of *B. lusitaniae* samples (country of origin, region, MLST alleles and STs) included in the present study are given in Table 2 and Table S2. An overview of the geographical distribution can be found in Figure 1. 

### 3.2. MLST, goeBURST and Phylogenetic Analyses of Borrelia Samples

#### 3.2.1. Multilocus Sequence Typing (MLST)

For 15 *B. lusitaniae* positive samples at least seven MLST genes provided good sequences and these samples were included in the MLST analysis conducted here (Table 2). These samples were complemented with 40 *B. lusitaniae* samples available via the *Borrelia* MLST database [(pubmlst.org/borrelia/ (11 February 2020)]. For several of the samples, *uvrA* did not yield a PCR product and, thus, we describe the isolates by seven genes, namely: *clpA, clpX, nifS*, *pepX, pyrG, recG* and *rplB*. Most samples of *B. lusitaniae* were from Portugal (*n* = 24), followed by Serbia (*n* = 15) and Algeria (*n* = 6) (Table 2). Among the 55 isolates there are 19 differing alleles of *clpA*, 19 different alleles of gene *clpX*, nine different alleles of gene *nifS*, 12 alleles of *pepX*, 14 alleles of *pyrG*, 10 alleles of *recG* genes and 17 *rplB* alleles.

#### 3.2.2. Clustering Analysis of *B. lusitaniae*

**Figure 2:**
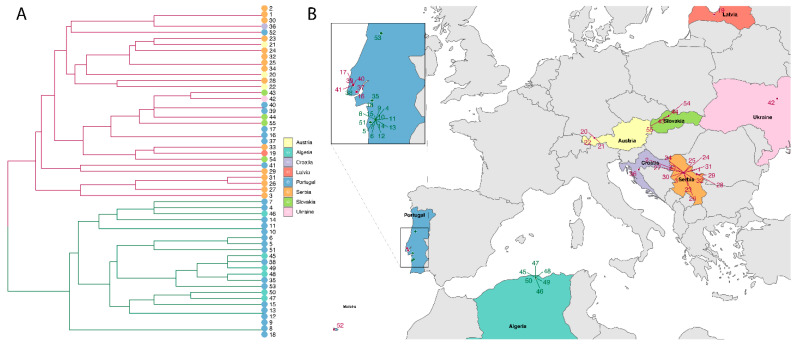
(**A**) Dendrogram of 55 isolates of *B. lusitaniae*. The dendrogram shows the similarity between isolates. Identical isolates are joined together on the same terminal branch, whereas a connection of isolates in the dendrogram further towards the left indicates less similarity (Gower, 1971, R). Color coding of terminal points is according to country (see legend), colors of the branches in the dendrogram specify the two clusters: cluster 1 = red, cluster 2 = green. (**B**) Geographical distribution of isolates belonging to cluster 1 and cluster 2. Color coding of countries corresponds to terminal points in 2A. Color coding of clusters is identical to the branch color in (**A**), cluster 1 = red, cluster 2 = green. The inlet shows the distribution of samples from mainland Portugal belonging to cluster 1 and cluster 2 in more detail. Most samples collected north of the river Tagus fell into cluster 1 while samples from south of the river Tagus fell into cluster 2.

Based on previous results for *B. lusitaniae* [33,59], we investigated the hypothesis that isolates may fall into two groups, one cluster of isolates from Portugal and Algeria and one cluster of isolates from Central/Eastern Europe. We assessed this hypothesis by using hierarchical clustering, numbering the isolates 1–55 (Table 2). In the dendogram in Figure 2A two different clusters are obvious (depicted in red and green branches). Within these groups identical isolates were identified: e.g., isolates 18, 8, 9, and 1, 2, 30 were identical.

The isolates (numbered 1–55) were plotted on a map (Figure 2B); numbers and color coding for cluster 1 (red) and cluster 2 (green) correspond in Figure 2A,B. Samples from Central/Eastern Europe all fell into cluster 1 while all samples from Algeria fell into cluster 2. Interestingly, isolates from Portugal were assigned to different clusters, a subset fell into cluster 1 and a subset into cluster 2 (inlet in Figure 2B). Six isolates from Mafra and Lisbon seem to be different from other Portuguese and North African isolates.

#### 3.2.3. goeBURST Analysis

**Figure 3:**
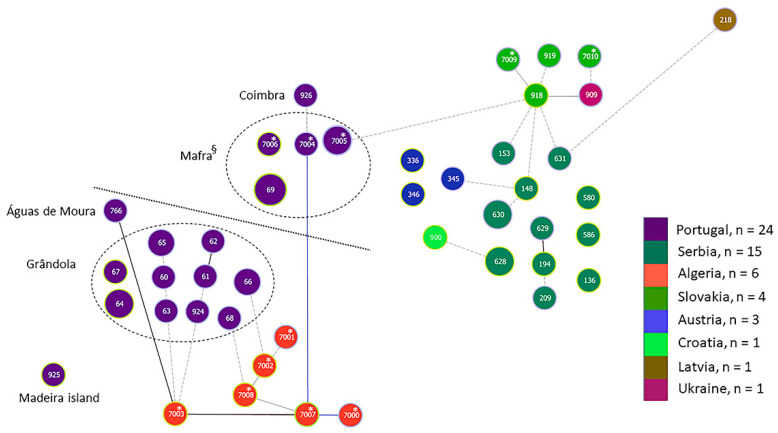
goeBURST diagram [83] of *B. lusitaniae* samples based on allele profile for seven MLST genes. The triple locus variant setting was chosen. Samples are color coded according to country of origin. For samples from Portugal, the region is indicated. The link color of edges are as follows: Link colors for goeBURST results: Black-Link drawn without recourse to tiebreak rules; Blue-Link drawn using tiebreak rule 1 (number of SLVs); Gray-Links drawn at DLV (darker gray) or TLV (lighter/dotted gray) if the groups are constructed at DLV/TLV level. * indicate STs derived from seven genes only with no correspondence to MLST database ST numbers. § includes one isolate from a human. Dotted line separates samples from the north and south of river Tagus in Portugal.

Since the cluster analysis was in line with previous results suggesting a population division of *B. lusitaniae*, we used goeBURST to obtain an additional view of the population using a different method. The goeBURST diagram also indicated a population division (Figure 3). Although there was one link based on tiebreak rule 1 between ST7-007* from Algeria and ST7-004* from Mafra in Portugal, several connections (one link without choice of tiebreak rules and several lower level edges; see figure legend Figure 3 for further information) were found between STs from Algeria and STs originating from south of the Tagus river in Portugal (Grândola and Águas de Moura), resulting in a clonal complex (CC) that included 17 STs (CC7-003). A bushy clonal complex (CC918) was also formed consisting of STs from Slovakia, Serbia, Austria, Latvia, Ukraine and one ST (ST7-005) from Mafra, Portugal.

When we used goeBURST on higher level settings (level 4, using four alleles to infer inter-isolate relationships; level 5, using 5 alleles and level 6, six alleles), the picture of the relationship within populations became more obvious (see Supplementary Figures S1–S3). Most of the Portuguese isolates clustered with North African isolates, except for some isolates from Lisbon (human), Mafra and Madeira island which clustered with those from Austria, Croatia, Latvia, Serbia and Slovakia.

#### 3.2.4. Sequence Analysis

**Figure 4:**
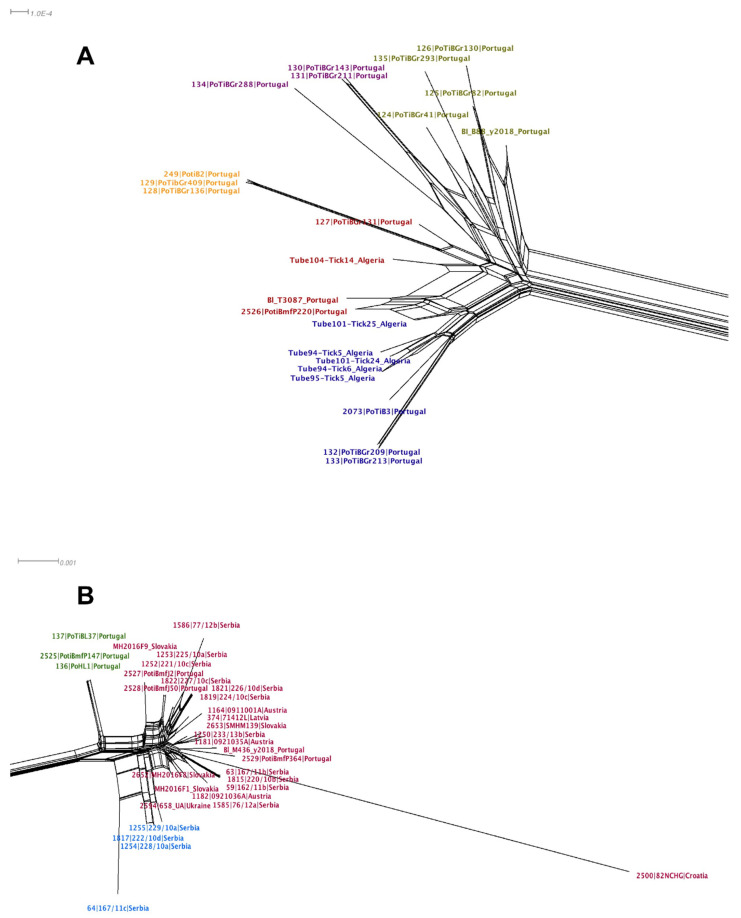
NeighborNet of the MLST alignment generated in Splitstree4 [73]. The net was yielded considering uncorrected P distance and equal angle display. Panel A shows the major group that includes the North African and some Portuguese isolates, whereas panel B shows the other major group containing most of the European isolates. The different color labels indicate the clusters found at the second level of clustering of the hierarchical population structure analysis (HPSA) using hierBAPS [77] (see Table S3). The scale bar shows the number of substitutions per site.

All analyses were conducted with concatenated sequences of seven MLST genes. As previously reported [33] we found evidence for recombination (*p* = 0.00125) in the *B. lusitaniae* dataset. Therefore, a NeighborNet [73] was constructed instead of a phylogeny. The net shows that there are two very well differentiated groups (see Figure 4A,B), which was confirmed by the first level of the HPSA (Table S3). One group consisted of Portuguese and Algerian isolates (Figure 4A); the other group combined isolates from Eastern Europe, Austria and Portugal (Figure 4B). Supplementary Figure S4 provides the whole network, where two major groups are well differentiated. Notably, we did not find any recombination events between these two groups using Gubbins; further suggesting that these are well differentiated groups that may not exchange DNA. We also computed the coefficient of differentiation (N_ST_) using MEGA X; the value of the coefficient was 0.71 (SE 0.04), which implies considerable levels of population differentiation between the two groups.

The second level of clustering using HPSA found clearly defined sub-clusters (see Table S3) within the two major groups, which suggests further geographic structuring. For instance, in major group 2 (Figure 4B) there is a cluster of Serbian isolates (blue label) and there is also a tight cluster of three Portuguese isolates (green label).

### 3.3. Analysis of Tick Samples

One hypothesis for the strong population division of *B. lusitaniae* was that this was due to vector association. To test this hypothesis, we investigated tick samples that originated from the same geographic region as the *B. lusitaniae* samples as well as from other regions. Ticks were identified to species status using morphological criteria [60,64,87]. Sequence data for the mitochondrial 16S rRNA gene and a nuclear gene, *trospA*, were used for corroboration. These loci were chosen because they showed in previous studies that there was a population division of *I. ricinus* between Europe and North Africa [61]. Because a new *Ixodes* species, *I. inopinatus*, was described originally in the Mediterranean region [60], samples from Germany that were identified as *I. inopinatus* morphologically and using molecular data (16S rRNA) were used as a control for *I. inopinatus*.

**Figure 5:**
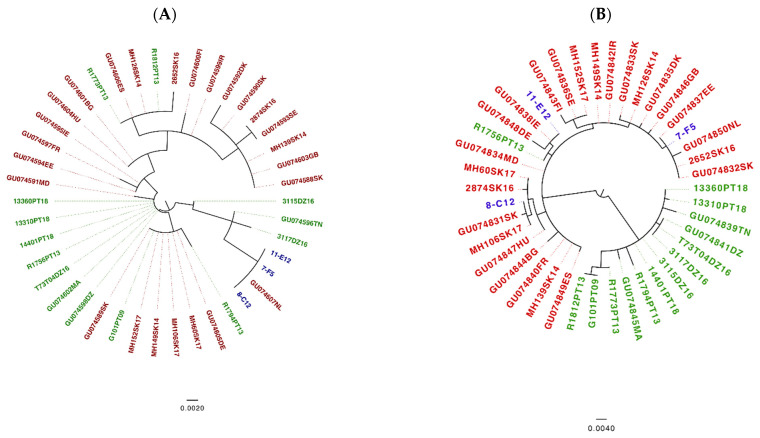
16S rRNA (**A**) and *trospA* (**B**) Maximum Likelihood phylogeny [86]. Phylogeny based on the 16S RNA gene (**A**). The model HK+I was used to construct the tree. Red labels mark *I. ricinus* non-Portuguese European sequences; green labels highlight *Ixodes* sequences of Portuguese and North African samples; and the blue labels denote *I. inopinatus* “reference” sequences from Germany. Phylogeny based on the *trospA* gene (**B**). The model K80+I was used to construct the tree. Red labels mark *I. ricinus* non-Portuguese European sequences; green labels highlight Portuguese and North African *Ixodes* sequences; and the blue labels denote *I. inopinatus* “reference” sequences. The scale bars show the number of substitutions per site.

Given that neither of the loci, 16S rRNA and *trospA*, showed recombination signals (16S rRNA *p* = 0.82 and *trospA p* = 0.13), we employed ML phylogenies to depict the evolutionary relationships for each locus. All ticks tested in Slovakia were determined as *I. ricinus* by sequencing both 16S rRNA and *trospA* genes and comparing the sequences via BLASTn [88] searches to sequences available in GenBank. In the 16S rRNA ML phylogeny and as far as *I. ricinus* samples are concerned, the Portuguese and North African samples were dispersed in several groups across the tree (Figure 5A, green color coding). Furthermore, the *I. inopinatus* samples (both from GenBank and our “reference samples”) grouped together with samples that were classified as *I. ricinus* morphologically. Figure 5B shows the phylogeny of *trospA*, which seems to agree with the MLST NeighborNet in Figure 4: Two clusters emerged in the *trospA* phylogeny according to the geographical distribution (whatever the tick species) as in the *B. lusitaniae* MLST NeighborNet. Interestingly, in the *trospA* phylogeny, with only one exception all the Portuguese samples cluster with the North African samples (Figure 5B, green color coding). The only exception being the Portuguese sample R1756PT13 collected in Mafra (north of the river Tagus), which clusters with other non-Portuguese samples from Europe (red color coding). Of note, the three *I. inopinatus* control (“reference”) samples from Germany (blue color coding) did not form a monophyletic group, as they were located on different branches far apart in the tree. Taken together these results suggest that unlike the 16S rRNA-based phylogeny, the *trospA* phylogeny is consistent with the MLST NeighborNet in that samples from North Africa and Portugal form a separate clade. Notably, neither in the *trospA* phylogeny nor in the 16S RNA phylogeny did the *I. inopinatus* samples form a monophyletic group.

## 4. Discussion

### 4.1. Population Structure of Borrelia lusitaniae

In this study, we have further evaluated the population structure of *B. lusitaniae* including additional samples from Croatia, Portugal, Slovakia, Ukraine as well as from Algeria using MLST. Consistent with previously reported data [59], our data clearly show a subdivision between North African isolates and isolates from Latvia, Serbia, Slovakia, and Austria. The population division in Portugal [33] was also corroborated. In the cluster analysis isolates from north of the Tagus river clustered together (cluster 1) and only two isolates fell into cluster 2 together with isolates from south of the Tagus river which is consistent with previously reported data by [33]. Southern Portuguese isolates showed a higher degree of genetic relatedness to isolates from Algeria using goeBURST than to isolates from Mafra. However, two Portuguese STs from south of the Tagus river clustered as singletons in the goeBURST diagram with TLV settings. When higher level settings were considered, two singletons from south of the Tagus river (ST67, ST64) clustered with Algerian samples while singletons from Mafra (ST69, ST7-006) and Madeira island (ST925) clustered with samples from Europe (Supplementary Information Figures S1–S3). Thus, of the two major clonal complexes, one (CC918) consisted of STs from Austria, Croatia, Slovakia, Serbia, Latvia, Ukraine and one Portuguese ST from Mafra, the other (CC7-007*) consisted primarily of STs from Algeria, southern Portugal and two isolates from Mafra and Coimbra. The division was strongly supported through the sequenced-based network analysis where most STs from Mafra (except one) clustered with STs from other European countries and all STs from Grândola and Algeria fell into one cluster together with PoTiB2 and PoTiB3 from Águas de Moura (south of river Tagus), corroborating data published using *ospA* as molecular marker [47,59]. In summary, our data support, and agree with, previously published data demonstrating a population division of *B. lusitaniae*.

Recombination analysis and an analysis of the degree of differentiation between the two *B. lusitaniae* clusters suggested considerable differentiation. Previous work on *Borrelia* has shown that intraspecific recombination rates are 50 times higher than interspecific recombination rates [89]. Furthermore, population genomics analyses of ocean bacteria investigating early events in population differentiation suggest that gradual separation of genes pools is accompanied by population-specific recombination [90,91]. It appears that the two major groups (populations) maybe in the early stages of speciation; each one on its own trajectory. First, both the population structure analysis and Neighbor net analysis clearly unveiled two very well-defined groups. Secondly, the absence of recombination events between these two groups may suggest that there has been no recent gene flow between the two groups. Clearly further investigations considering whole genome sequences are needed to establish if there is gene flow between the two major populations.

The observed population division of *B. lusitaniae* did not seem to follow climate or vegetation zones known for Europe [92]. North Portugal may be influence by the Atlantic region while countries such as Italy, Spain and Algeria belong to the sub-Mediterranean or Mediterranean region. Although one could speculate that the division of Portuguese *B. lusitaniae* populations may follow the pattern of floristic regions or may be influenced by a geographic barrier (river Tagus), it would not explain the observed clustering of *B. lusitaniae* STs from North Portugal with STs from other European regions.

### 4.2. Host and Vector Associations

Previous work has suggested that host association is a key factor for driving diversification and speciation in *Borrelia* [6,14]. Subsequently it was suggested that vector associations may also drive diversification and ultimately speciation in *Borrelia* [11]. In view of the latest described *Ixodes* species (*I. inopinatus*) and its geographic distribution in North Africa, southern Spain and Portugal [60], it was a very attractive hypothesis to investigate whether the association with different vector species may be responsible for the observed population division. However, our data do not unambiguously support the hypothesis that an association with *I. inopinatus* is responsible for the observed population division of *B. lusitaniae*. We added three sample of *I. inopinatus* as reference because these were morphologically identified as *I. inopinatus* and their 16S rRNA sequences were identical to samples designated *I. inopinatus* in GenBank. Surprisingly, the *I. inopinatus* sequences from GenBank (GenBank accession numbers for 16S rRNA/*trospA*, respectively: GU074596TN/GU074839TN; GU074598DZ/GU074841DZ; GU074602MA/GU074845MA) and our own *I. inopinatus* reference samples did not form a monophyletic group. These data call for good reference sequences for this tick species and currently we cannot confirm the hypothesis of *I. inopinatus* association being the reason for the population division of *B. lusitaniae*. Intriguingly, in the *trospA* tree sequences clustered according to geographical origin: a divergent clade contained (with one exception) all the Portuguese and North African samples, samples designated as *I. inopinatus* in GenBank and one sample that the 16S rRNA BLAST search identified as *I. inopinatus* (3117DZ16). A separation between North African and Eurasian *I. ricinus* was also found by [61] and [93] using concatenated sequences of four mitochondrial and nuclear genes or 125 single nucleotide polymorphisms, respectively, although none of the studies included tick samples from Portugal. Thus, the *B. lusitaniae* population structure obtained by MLST seems to match the population structure of *Ixodes* using the *trospA* gene. Although these data are suggestive of co-evolution between *B. lusitaniae* and its vector, the drivers for this process remain to be elucidated.

Regarding host association, in the various countries were *B. lusitaniae* has been described, several lizard species have been suggested as reservoir hosts. In Slovakia, Germany and Italy green lizards (*L*. *viridis*) [46], sand lizards (*L. agilis*) [43] and common wall lizards (*Podarcis muralis*) [37,43,44] were described as reservoir hosts. However, these species do not occur on the Iberian Peninsula or in North Africa (see also https://www.Eurolizards.com). In Portugal and Spain, *L. schreiberi* is commonly found but its role as reservoir for *B. lusitaniae* cannot be confirmed with current data, although feeding *I. ricinus* nymphs have been found on this host [38]. *Psammodromus algirus*, the Algerian lizard, is widespread in North Africa (Algeria, Morocco, Tunisia) and on the Iberian Peninsula but its distribution in Italy or other countries is very restricted. The species has been shown to be divided into differentiated populations in East and West Iberia and North Africa. Furthermore, this species also shows some lineage differentiation in its western range in Iberia, between northern and southern populations, but miscegenation and relatively small sample size has precluded any taxonomic divisions [94,95]. The species has been identified as reservoir host for *B. lusitaniae* in Tunisia [36,42,96] and Portugal [38]. Given the different geographic distributions of potential reservoir hosts for *B. lusitaniae*, it remains to be investigated how narrow the niche for reservoir hosts of *B. lusitaniae* is and serum sensitivity or transmission experiments could help solving this question.

## 5. Conclusions

In this study, we have expanded investigations on the population structure of *B. lusitaniae* and confirmed a population division separating samples from southern Portugal and Algeria from samples from northern Portugal and other European countries. Molecular analyses of morphologically identified *Ixodes* samples (encompassing *I. ricinus* and *I. inopinatus*) acquired from the same geographical regions as *Borrelia* samples showed that the two loci investigated, i.e., 16S rRNA and *trospA*, were phylogenetically incongruous also with respect to clustering of *Ixodes* species. One clade in the *trospA* phylogeny consisted mostly of tick samples from North Africa and Portugal and mirrored the population division found in *Borrelia*. These data suggest that some co-evolution between *Ixodes* and *B. lusitaniae* populations may have occurred which warrants further investigation.

## Figures and Tables

**Table 1:** Tick samples included in this study.

Tick AN for NCBI_16S rRNA	Tick AN for NCBI_*trospA*	Tick Species (16S rRNA Based ID)	Year	Stage/Sex	*B. lusitaniae* Infection Status	MLST *B. lusitaniae*		Region Specific †	Reference
GU074596TN	GU074839TN	*Ixodes inopinatus #*		F	unknown			TN, Jbel el Jouza	[61]
GU074598DZ ‡	GU074841DZ	*Ixodes inopinatus #*		F	unknown			DZ, El Tarf	[61]
GU074602MA	GU074845MA	*Ixodes inopinatus #*		M	unknown			MA, Taza	[61]
GU074597FR	GU074840FR ‡	*Ixodes ricinus*		F	unknown			FR, Forêt de Chizé	[61]
GU074606ES ‡	GU074849ES	*Ixodes ricinus*		F	unknown			ES, Otxandio	[61]
GU074595IE ‡	GU074838IE	*Ixodes ricinus*		F	unknown			IE, Cork, Killarney National park	[61]
GU074603GB	GU074846GB	*Ixodes ricinus*		N	unknown			UK	[61]
GU074607NL	GU074850NL	*Ixodes ricinus*		F	unknown			NL	[61]
GU074605DE ‡	GU074848DE	*Ixodes ricinus*		N	unknown			DE, Munich, English garden	[61]
GU074599IR	GU074842IR	*Ixodes ricinus*		F	unknown			IR, Mazendaran province	[61]
GU074592DK	GU074835DK	*Ixodes ricinus*		F	unknown			DK, Grib Skov Forest	[61]
GU074593SE ‡	GU074836SE	*Ixodes ricinus*		F	unknown			SE, Alsike	[61]
GU074588SK	GU074831SK	*Ixodes ricinus*		F	unknown			SK, Železná Studnička	[61]
GU074589SK ‡	GU074832SK ‡	*Ixodes ricinus*		F	unknown			SK, Vel’ký Lom	[61]
GU074590SK	GU074833SK	*Ixodes ricinus*		F	unknown			SK, Malá Lehota	[61]
GU074594EE ‡	GU074837EE ‡	*Ixodes ricinus*		F	unknown			EE, Tartumaa	[61]
GU074600FI	GU074843FI	*Ixodes ricinus*		F	unknown			FI, Turku archipelago	[61]
GU074604HU	GU074847HU	*Ixodes ricinus*		F	unknown			HU, Mátrafüred forest	[61]
GU074591MD	GU074834MD ‡	*Ixodes ricinus*		F	unknown			MD	[61]
GU074601BG	GU074844BG	*Ixodes ricinus*		F	unknown			BG, Sofia	[61]
13310PT18 ‡	13310PT18	*Ixodes ricinus*	2018	F	positive		south Tagus	PT, Santiago do Cacém	This study
13360PT18 ‡	13360PT18	*Ixodes ricinus*	2018	F	positive		south Tagus	PT, Grândola	This study
14401PT18 ‡	14401PT18	*Ixodes ricinus*	2018	F	positive		south Tagus	PT, Santiago do Cacém	This study
G101PT09 ‡	G101PT09	*Ixodes ricinus*	2009	N	unknown		north Tagus	PT, Gerês	This study
R1756PT13 ‡	R1756PT13	*Ixodes ricinus*	2013	N	positive		north Tagus	PT, Mafra	This study
R1773PT13 ‡	R1773PT13	*Ixodes ricinus*	2013	N	positive		north Tagus	PT, Mafra	This study
R1812PT13 ‡	R1812PT13	*Ixodes ricinus*	2013	N	negative		north Tagus	PT, Mafra	This study
R1794PT13	R1794PT13	*Ixodes ricinus*	2013	N	negative		north Tagus	PT, Mafra	This study
3117DZ16	3117DZ16	*Ixodes inopinatus*	2016		positive	yes: ID 3117		DZ, Kabylie	This study
T73T04DZ16 ‡	T73T04DZ16	*Ixodes ricinus*	2016		positive			DZ, Kabylie	This study
3115DZ16	3115DZ16	*Ixodes ricinus*	2016		positive			DZ, Kabylie	This study
MH152SK17 ‡	MH152SK17	*Ixodes ricinus*	2017	M	positive			SK, Martinské hole	This study
MH149SK14 ‡	MH149SK14	*Ixodes ricinus*	2014	M	positive			SK, Martinské hole	This study
MH139SK14	MH139SK14	*Ixodes ricinus*	2014	M	positive	yes: ID 2653		SK, Martinské hole	This study
MH106SK17 ‡	MH106SK17	*Ixodes ricinus*	2017	F	positive			SK, Martinské hole	This study
MH60SK17 ‡	MH60SK17	*Ixodes ricinus*	2017	F	positive			SK, Martinské hole	This study
2874SK16 ‡	2874SK16	*Ixodes ricinus*	2016	F	positive			SK, Martinské hole	This study
MH126SK14 ‡	MH126SK14	*Ixodes ricinus*	2014	F	positive			SK, Martinské hole	This study
2652SK16	2652SK16	*Ixodes ricinus*	2016	F	positive	yes: ID 2652		SK, Martinské hole	This study
11-E12	11-E12	*Ixodes inopinatus*	2018	N	negative			DE, Starnberg	This study
7-F5	7-F5	*Ixodes inopinatus*	2018	N	negative			DE, Starnberg	This study
8-C12	8-C12	*Ixodes inopinatus*	2018	N	negative			DE, Starnberg	This study

AN = accession number, M = male, F = female, N = nymh, † the two-letter country code (ISO 3166) is given, ‡ Sequence detected in more than one country., # designated *I. ricinus* in Noureddine et al. 2011 [61] but now designated *I. inopinatus* in GenBank.

**Table 2:** *Borrelia lusitaniae* samples included in this study for which seven MLST genes were available.

Sample No.	Sample ID	Strain	Genospecies	Country	Area	Continent	ST	Year of Collection	Biological Source	Tick Code	Reference
1	59	162/11b	*Borrelia lusitaniae*	Serbia	Belgrade-Titov gaj	Europe	628	2011	*Ixodes ricinus*		MLST DB
2	63	167/11b	*Borrelia lusitaniae*	Serbia	Belgrade-Kosutnjak	Europe	628	2011	*Ixodes ricinus*		MLST DB
3	64	167/11c	*Borrelia lusitaniae*	Serbia	Belgrade-Kosutnjak	Europe	136	2011	*Ixodes ricinus*		MLST DB
4	124	PoTiBGr41	*Borrelia lusitaniae*	Portugal	Grândola	Europe	60	2002	*Ixodes ricinus*		MLST DB
5	125	PoTiBGr82	*Borrelia lusitaniae*	Portugal	Grândola	Europe	61	2002	*Ixodes ricinus*		MLST DB
6	126	PoTiBGr130	*Borrelia lusitaniae*	Portugal	Grândola	Europe	62	2003	*Ixodes ricinus*		MLST DB
7	127	PoTiBGr131	*Borrelia lusitaniae*	Portugal	Grândola	Europe	63	2003	*Ixodes ricinus*		MLST DB
8	128	PoTiBGr136	*Borrelia lusitaniae*	Portugal	Grândola	Europe	64	2003	*Ixodes ricinus*		MLST DB
9	129	PoTibGr409	*Borrelia lusitaniae*	Portugal	Grândola	Europe	64	2003	*Ixodes ricinus*		MLST DB
10	130	PoTiBGr143	*Borrelia lusitaniae*	Portugal	Grândola	Europe	65	2003	*Ixodes ricinus*		MLST DB
11	131	PoTiBGr211	*Borrelia lusitaniae*	Portugal	Grândola	Europe	65	2003	*Ixodes ricinus*		MLST DB
12	132	PoTiBGr209	*Borrelia lusitaniae*	Portugal	Grândola	Europe	66	2003	*Ixodes ricinus*		MLST DB
13	133	PoTiBGr213	*Borrelia lusitaniae*	Portugal	Grândola	Europe	66	2003	*Ixodes ricinus*		MLST DB
14	134	PoTiBGr288	*Borrelia lusitaniae*	Portugal	Grândola	Europe	67	2003	*Ixodes ricinus*		MLST DB
15	135	PoTiBGr293	*Borrelia lusitaniae*	Portugal	Grândola	Europe	68	2003	*Ixodes ricinus*		MLST DB
16	136	PoHL1	*Borrelia lusitaniae*	Portugal	Lisbon	Europe	69	2002	*human*		MLST DB
17	137	PoTiBL37	*Borrelia lusitaniae*	Portugal	Mafra	Europe	69	1999	*Ixodes ricinus*		MLST DB
18	249	PotiB2	*Borrelia lusitaniae*	Portugal		Europe	64		*Ixodes ricinus*		MLST DB
19	374	71412L	*Borrelia lusitaniae*	Latvia		Europe	218	2007	*Ixodes ricinus*		MLST DB
20	1164	0911001A	*Borrelia lusitaniae*	Austria	North-Tirol	Europe	336	2009	*Ixodes ricinus*		MLST DB
21	1181	0921035A	*Borrelia lusitaniae*	Austria	North-Tirol	Europe	345	2009	*Ixodes ricinus*		MLST DB
22	1182	0921036A	*Borrelia lusitaniae*	Austria	North-Tirol	Europe	346	2009	*Ixodes ricinus*		MLST DB
23	1250	233/13b	*Borrelia lusitaniae*	Serbia	Belgrade-Avala	Europe	148	2013	*Ixodes ricinus*		MLST DB
24	1252	221/10c	*Borrelia lusitaniae*	Serbia	Belgrade-Titov gaj	Europe	153	2013	*Ixodes ricinus*		MLST DB
25	1253	225/10a	*Borrelia lusitaniae*	Serbia	Belgrade-Titov gaj	Europe	630	2013	*Ixodes ricinus*		MLST DB
26	1254	228/10a	*Borrelia lusitaniae*	Serbia	Belgrade-Titov gaj	Europe	194	2013	*Ixodes ricinus*		MLST DB
27	1255	229/10a	*Borrelia lusitaniae*	Serbia	Belgrade-Kosutnjak	Europe	209	2013	*Ixodes ricinus*		MLST DB
28	1585	76/12a	*Borrelia lusitaniae*	Serbia	Eastern Serbia-Dobra (Djerdap Gorge)	Europe	580	2012	*Ixodes ricinus*		MLST DB
29	1586	77/12b	*Borrelia lusitaniae*	Serbia	Eastern Serbia-Dobra (Djerdap Gorge)	Europe	586	2012	*Ixodes ricinus*		MLST DB
30	1815	220/10b	*Borrelia lusitaniae*	Serbia	Belgrade-Kosutnjak	Europe	628	2010	*Ixodes ricinus*		MLST DB
31	1817	222/10d	*Borrelia lusitaniae*	Serbia	Belgrade-Titov gaj	Europe	629	2010	*Ixodes ricinus*		MLST DB
32	1819	224/10c	*Borrelia lusitaniae*	Serbia	Belgrade-Titov gaj	Europe	630	2010	*Ixodes ricinus*		MLST DB
33	1821	226/10d	*Borrelia lusitaniae*	Serbia	Belgrade-Titov gaj	Europe	631	2010	*Ixodes ricinus*		MLST DB
34	1822	227/10c	*Borrelia lusitaniae*	Serbia	Belgrade-Titov gaj	Europe	630	2010	*Ixodes ricinus*		MLST DB
35	2073	PoTiB3	*Borrelia lusitaniae*	Portugal		Europe	766	1993	*Ixodes ricinus*		MLST DB
36	2500	82NCHG	*Borrelia lusitaniae*	Croatia	Grabovac	Europe	900	2011	*Ixodes ricinus*		This study
37	2525	PotiBmfP147	*Borrelia lusitaniae*	Portugal	Mafra	Europe	7-011	2003	*Ixodes ricinus*		MLST DB
38	2526	PotiBmfP220	*Borrelia lusitaniae*	Portugal	Mafra	Europe	7-004	2003	*Ixodes ricinus*		MLST DB
39	2527	PotiBmfJ2	*Borrelia lusitaniae*	Portugal	Mafra	Europe	7-005	2001	*Ixodes ricinus*		MLST DB
40	2528	PotiBmfJ50	*Borrelia lusitaniae*	Portugal	Mafra	Europe	7-005	2003	*Ixodes ricinus*		MLST DB
41	2529	PotiBmfP364	*Borrelia lusitaniae*	Portugal	Mafra	Europe	7-006	2003	*Ixodes ricinus*		MLST DB
42	2594	658 UA	*Borrelia lusitaniae*	Ukraine	Kyiv, M.M. Gryshko Nat. Bot. Garden	Europe	909	2015	*Ixodes ricinus*		This study
43	2652	MH2016F8	*Borrelia lusitaniae*	Slovakia	Martinské hole mountain	Europe	918	2016	*Ixodes ricinus*	2652SK16	This study
44	2653	SMHM139	*Borrelia lusitaniae*	Slovakia	Martinské hole mountain	Europe	919	2013	*Ixodes ricinus*	MH139SK14	This study
52	3126	PoTiB10/M436	*Borrelia lusitaniae*	Portugal	Madeira	Europe	925	2009	*Ixodes ricinus*		This study
53	3127	PoTiB11/T3087	*Borrelia lusitaniae*	Portugal	Coimbra	Europe	926	2014			This study
51	3125	PoTiB9/B88	*Borrelia lusitaniae*	Portugal	Santiago do Cacém	Europe	924	2009	*Dermacentor* *marginatum*		This study
45	3114	Tube101-Tick25/run1-2	*Borrelia lusitaniae*	Algeria	Kabylie	Africa	7-000	2016	*Ixodes ricinus*		This study
46	3117	Tube104-Tick14/run1-5	*Borrelia lusitaniae*	Algeria	Kabylie	Africa	7-001	2016	*Ixodes ricinus*	3117DZ16	This study
47	3118	Tube94-Tick5/run1-6	*Borrelia lusitaniae*	Algeria	Kabylie	Africa	7-002	2016	*Ixodes ricinus*		This study
48	3120	Tube101-Tick24/run1-11	*Borrelia lusitaniae*	Algeria	Kabylie	Africa	7-003	2016	*Ixodes ricinus*		This study
49	3119	Tube 95-Tick5/run1-9	*Borrelia lusitaniae*	Algeria	Kabylie	Africa	7-007	2016	*Ixodes ricinus*		This study
50	3123	Tube94-tick6/run2-7	*Borrelia lusitaniae*	Algeria	Kabylie	Africa	7-008	2016	*Ixodes ricinus*		This study
54	2873	MH2016F1	*Borrelia lusitaniae*	Slovakia	Martinské hole mountain	Europe	7-009	2016	*Ixodes ricinus*		This study
55	2874	MH2016F9	*Borrelia lusitaniae*	Slovakia	Martinské hole mountain	Europe	7-010	2016	*Ixodes ricinus*		This study

7-0XX = provisional ST number. MLST DB – Multilocus sequence typing database www.pubmlst.org/borrelia/ (accessed on 11 February 2020).

## Data Availability

MLST data: All MLST sequence data are available from the MLST database at https://pubmlst.org/borrelia/ (accessed on 23 April 2021) via isolate names. GenBank Accession numbers: Tick sequences obtained and used in this study were submitted to GenBank under the accession numbers MW017342-MW017360 and MW287230-MW287232 (mitochondrial 16S rRNA locus) and MW379826-MW379844 and MW344866–MW344868 (nuclear gene *trospA*).

## Supplementary Materials

The following are available online at https://www.mdpi.com/article/10.3390/microorganisms9050933/s1, Table S1: *Ixodes* tick samples included in this study including information on processing of samples, Table S2: *Borrelia* samples included in this study, sequence type (ST) number (if available), allele numbers of the seven genes included for analyses and details of sample processing, Table S3: Detailed results of Hierarchical BAPS analyses at levels 1 and 2, Supplementary Methods, Figure S1: Phyloviz diagram [83] of *B. lusitaniae* samples based on allele profile for seven MLST genes (level 4), Figure S2: Phyloviz diagram of *B. lusitaniae* samples based on allele profiles for seven MLST genes (level 5), Figure S3: Phyloviz diagram of *B. lusitaniae* samples based on allele profiles for seven MLST genes (level 6), Figure S4: NeighborNet of the MLST alignment generated in Splitstree4.

## References

[1] Kurtenbach K., Hoen A.G., Bent S.J., Vollmer S.A., Ogden N.H., Margos G., Robinson D.A., Falush D., Feil E.J. (2010). Population biology of lyme borreliosis spirochetes. Bacterial Population Genetics in Infectious Disease.

[2] Margos G., Vollmer S.A., Ogden N.H., Fish D. (2011). Population genetics, taxonomy, phylogeny and evolution of *Borrelia burgdorferi* sensu lato. Infect. Genet. Evol..

[3] Rizzoli A., Tagliapietra V., Cagnacci F., Marini G., Arnoldi D., Rosso F., Rosa R. (2019). Parasites and wildlife in a changing world: The vector-host-pathogen interaction as a learning case. Int. J. Parasitol. Parasites Wildl..

[4] Frank S.A. (2002). Immunology and Evolution of Infectious Disease.

[5] Jongejan F., Uilenberg G. (2004). The global importance of ticks. Parasitology.

[6] Kurtenbach K., Hanincova K., Tsao J.I., Margos G., Fish D., Ogden N.H. (2006). Fundamental processes in the evolutionary ecology of Lyme borreliosis. Nat. Rev. Microbiol..

[7] Telford S.R., Goethert H.K. (2004). Emerging tick-borne infections: Rediscovered and better characterized, or truly ‘new’?. Parasitology.

[8] Medlock J.M., Hansford K.M., Bormane A., Derdakova M., Estrada-Peña A., George J.-C., Golovjona I., Jaenson T.G.T., Jensen J.-K., Jensen P.M. (2013). Driving forces for changes in geographical distribution of *Ixodes ricinus* ticks in Europe. Parasites Vectors.

[9] Rizzoli A., Hauffe H.C., Carpi G., Vourc’h G.I., Neteler M., Rosà R. (2011). Lyme borreliosis in Europe. Euro Surveill.

[10] Becker N.S., Margos G., Blum H., Krebs S., Graf A., Lane R.S., Castillo-Ramirez S., Sing A., Fingerle V. (2016). Recurrent evolution of host and vector association in bacteria of the *Borrelia burgdorferi* sensu lato species complex. BMC Genom..

[11] Margos G., Fingerle V., Reynolds S.E. (2019). *Borrelia bavariensis*: Vector Switch, Niche Invasion, and Geographical Spread of a Tick-Borne Bacterial Parasite. Front. Ecol. Evol..

[12] Vollmer S.A., Bormane A., Dinnis R.E., Seelig F., Dobson A.D., Aanensen D.M., James M.C., Donaghy M., Randolph S.E., Feil E.J. (2011). Host migration impacts on the phylogeography of Lyme Borreliosis spirochaete species in Europe. Environ. Microbiol..

[13] Vollmer S.A., Feil E.J., Chu C.Y., Raper S.L., Cao W.C., Kurtenbach K., Margos G. (2013). Spatial spread and demographic expansion of Lyme borreliosis spirochaetes in Eurasia. Infect. Genet. Evol..

[14] Ogden N.H., Mechai S., Margos G. (2013). Changing geographic ranges of ticks and tick-borne pathogens: Drivers, mechanisms and consequences for pathogen diversity. Front. Cell. Infect. Microbiol..

[15] Tsao J.I. (2009). Reviewing molecular adaptations of Lyme borreliosis spirochetes in the context of reproductive fitness in natural transmission cycles. Vet. Res..

[16] Hanincova K., Kurtenbach K., Diuk-Wasser M., Brei B., Fish D. (2006). Epidemic spread of Lyme borreliosis, northeastern United States. Emerg. Infect. Dis..

[17] Eisen L. (2020). Vector competence studies with hard ticks and *Borrelia burgdorferi* sensu lato spirochetes: A review. Ticks Tick Borne Dis..

[18] Diuk-Wasser M.A., Gatewood A.G., Cortinas M.R., Yaremych-Hamer S., Tsao J., Kitron U., Hickling G., Brownstein J.S., Walker E., Piesman J. (2006). Spatiotemporal patterns of host-seeking *Ixodes scapularis* nymphs (Acari: *Ixodidae*) in the United States. J. Med. Entomol..

[19] Mechai S., Margos G., Feil E.J., Lindsay L.R., Ogden N.H. (2015). Complex population structure of *Borrelia burgdorferi* in southeastern and south central Canada as revealed by phylogeographic analysis. Appl. Environ. Microbiol..

[20] Ogden N.H., Lindsay L.R., Hanincova K., Barker I.K., Bigras-Poulin M., Charron D.F., Heagy A., Francis C.M., O’Callaghan C.J., Schwartz I. (2008). Role of migratory birds in introduction and range expansion of *Ixodes scapularis* ticks and of *Borrelia burgdorferi* and *Anaplasma phagocytophilum* in Canada. Appl. Environ. Microbiol..

[21] Ogden N.H., Margos G., Aanensen D.M., Drebot M.A., Feil E.J., Hanincova K., Schwartz I., Tyler S., Lindsay L.R. (2011). Investigation of genotypes of *Borrelia burgdorferi* in *Ixodes scapularis* ticks collected during surveillance in Canada. Appl. Environ. Microbiol..

[22] Lane R.S., Loye J.E. (1991). Lyme disease in California: Interrelationship of ixodid ticks (Acari), rodents, and *Borrelia burgdorferi*. J. Med. Entomol..

[23] Girard Y.A., Travinsky B., Schotthoefer A., Fedorova N., Eisen R.J., Eisen L., Barbour A.G., Lane R.S. (2009). Population structure of the lyme borreliosis spirochete *Borrelia burgdorferi* in the western black-legged tick (*Ixodes pacificus*) in Northern California. Appl. Environ. Microbiol..

[24] Tyler S., Tyson S., Dibernardo A., Drebot M., Feil E.J., Graham M., Knox N.C., Lindsay L.R., Margos G., Mechai S. (2018). Whole genome sequencing and phylogenetic analysis of strains of the agent of Lyme disease *Borrelia burgdorferi* from Canadian emergence zones. Sci. Rep..

[25] Walter K.S., Carpi G., Caccone A., Diuk-Wasser M.A. (2017). Genomic insights into the ancient spread of Lyme disease across North America. Nat. Ecol. Evol..

[26] Margos G., Tsao J.I., Castillo-Ramirez S., Girard Y.A., Hamer S.A., Hoen A.G., Lane R.S., Raper S.L., Ogden N.H. (2012). Two boundaries separate *Borrelia burgdorferi* populations in North America. Appl. Environ. Microbiol..

[27] Castillo-Ramirez S., Fingerle V., Jungnick S., Straubinger R.K., Krebs S., Blum H., Meinel D.M., Hofmann H., Guertler P., Sing A. (2016). Trans-Atlantic exchanges have shaped the population structure of the Lyme disease agent *Borrelia burgdorferi* sensu stricto. Sci. Rep..

[28] Gomez-Diaz E., Boulinier T., Sertour N., Cornet M., Ferquel E., McCoy K.D. (2011). Genetic structure of marine *Borrelia garinii* and population admixture with the terrestrial cycle of Lyme borreliosis. Environ. Microbiol..

[29] Comstedt P., Jakobsson T., Bergstrom S. (2011). Global ecology and epidemiology of *Borrelia garinii* spirochetes. Infect. Ecol. Epidemiol..

[30] Munro H.J., Ogden N.H., Mechai S., Lindsay L.R., Robertson G.J., Whitney H., Lang A.S. (2019). Genetic diversity of *Borrelia garinii* from *Ixodes uriae* collected in seabird colonies of the northwestern Atlantic Ocean. Ticks Tick Borne Dis..

[31] Norte A.C., Margos G., Becker N.S., Albino Ramos J., Nuncio M.S., Fingerle V., Araujo P.M., Adamik P., Alivizatos H., Barba E. (2020). Host dispersal shapes the population structure of a tick-borne bacterial pathogen. Mol. Ecol..

[32] Mtierova Z., Derdakova M., Chvostac M., Didyk Y.M., Mangova B., Rusnakova Taragelova V., Selyemova D., Sujanova A., Vaclav R. (2020). Local Population Structure and Seasonal Variability of *Borrelia garinii* Genotypes in *Ixodes ricinus* Ticks, Slovakia. Int. J. Environ. Res. Public Health.

[33] Vitorino L.R., Margos G., Feil E.J., Collares-Pereira M., Ze-Ze L., Kurtenbach K. (2008). Fine-scale Phylogeographic Structure of *Borrelia lusitaniae* Revealed by Multilocus Sequence Typing. PLoS ONE.

[34] Núncio M.S., Péter O., Alves M.J., Bacellar F., Filipe A.R. (1993). Isolamento e caracterização de borrélias de *Ixodes ricinus* L. em Portugal. Revista Portuguesa Doenças Infecciosas.

[35] Le Fleche A., Postic D., Girardet K., Peter O., Baranton G. (1997). Characterization of *Borrelia lusitaniae* sp. nov. by 16S ribosomal DNA sequence analysis. Int. J. Syst. Bacteriol..

[36] Dsouli N., Younsi-Kabachii H., Postic D., Nouira S., Gern L., Bouattour A. (2006). Reservoir role of lizard *Psammodromus algirus* in transmission cycle of *Borrelia burgdorferi* sensu lato (*Spirochaetaceae*) in Tunisia. J. Med. Entomol..

[37] Amore G., Tomassone L., Grego E., Ragagli C., Bertolotti L., Nebbia P., Rosati S., Mannelli A. (2007). *Borrelia lusitaniae* in immature *Ixodes ricinus* (Acari: *Ixodidae*) feeding on common wall lizards in Tuscany, central Italy. J. Med. Entomol..

[38] Norte A.C., Alves da Silva A., Alves J., da Silva L.P., Nuncio M.S., Escudero R., Anda P., Ramos J.A., Lopes de Carvalho I. (2015). The importance of lizards and small mammals as reservoirs for *Borrelia lusitaniae* in Portugal. Environ. Microbiol. Rep..

[39] Baptista S., Quaresma A., Aires T., Kurtenbach K., Santos-Reis M., Nicholson M., Collares-Pereira M. (2004). Lyme borreliosis spirochetes in questing ticks from mainland Portugal. Int. J. Med. Microbiol..

[40] Norte A.C., Ramos J.A., Gern L., Nuncio M.S., Lopes de Carvalho I. (2013). Birds as reservoirs for *Borrelia burgdorferi* s.l. in Western Europe: Circulation of *B. turdi* and other genospecies in bird-tick cycles in Portugal. Environ. Microbiol..

[41] De Carvalho I.L., Milhano N., Santos A.S., Almeida V., Barros S.C., De Sousa R., Nuncio M.S. (2008). Detection of *Borrelia lusitaniae*, *Rickettsia* sp. IRS3, *Rickettsia monacensis*, and *Anaplasma phagocytophilum* in *Ixodes ricinus* collected in Madeira Island, Portugal. Vector Borne Zoonotic Dis..

[42] Younsi H., Postic D., Baranton G., Bouattour A. (2001). High prevalence of *Borrelia lusitaniae* in *Ixodes ricinus* ticks in Tunisia. Eur. J. Epidemiol..

[43] Richter D., Matuschka F.R. (2006). Perpetuation of the Lyme disease spirochete *Borrelia lusitaniae* by lizards. Appl. Environ. Microbiol..

[44] Ragagli C., Bertolotti L., Giacobini M., Mannelli A., Bisanzio D., Amore G., Tomassone L. (2011). Transmission dynamics of *Borrelia lusitaniae* and *Borrelia afzelii* among *Ixodes ricinus*, lizards, and mice in Tuscany, central Italy. Vector Borne Zoonotic Dis..

[45] De Sousa R., Lopes de Carvalho I., Santos A.S., Bernardes C., Milhano N., Jesus J., Menezes D., Nuncio M.S. (2012). Role of the lizard *Teira dugesii* as a potential host for *Ixodes ricinus* tick-borne pathogens. Appl. Environ. Microbiol..

[46] Majlathova V., Majlath I., Derdakova M., Vichova B., Pet’ko B. (2006). *Borrelia lusitaniae* and green lizards (*Lacerta viridis*), Karst Region, Slovakia. Emerg. Infect. Dis..

[47] De Carvalho I.L., Zeidner N., Ullmann A., Hojgaard A., Amaro F., Ze-Ze L., Alves M.J., de Sousa R., Piesman J., Nuncio M.S. (2010). Molecular characterization of a new isolate of *Borrelia lusitaniae* derived from *Apodemus sylvaticus* in Portugal. Vector Borne Zoonotic Dis..

[48] Sarih M., Jouda F., Gern L., Postic D. (2003). First isolation of *Borrelia burgdorferi* sensu lato from *Ixodes ricinus* ticks in Morocco. Vector Borne Zoonotic Dis..

[49] Zhioua E., Bouattour A., Hu C.M., Gharbi M., Aeschliman A., Ginsberg H.S., Gern L. (1999). Infection of *Ixodes ricinus* (Acari: *Ixodidae*) by *Borrelia burgdorferi* sensu lato in North Africa. J. Med. Entomol..

[50] Bertolotti L., Tomassone L., Tramuta C., Grego E., Amore G., Ambrogi C., Nebbia P., Mannelli A. (2006). *Borrelia lusitaniae* and spotted fever group rickettsiae in *Ixodes ricinus* (Acari: *Ixodidae*) in Tuscany, central Italy. J. Med. Entomol..

[51] Taragelova V.R., Mahrikova L., Selyemova D., Vaclav R., Derdakova M. (2016). Natural foci of *Borrelia lusitaniae* in a mountain region of Central Europe. Ticks Tick Borne Dis..

[52] Wodecka B., Skotarczak B. (2005). First isolation of Borrelia lusitaniae DNA from Ixodes ricinus ticks in Poland. Scand. J. Infect. Dis..

[53] Okeyo M., Hepner S., Rollins R.E., Hartberger C., Straubinger R.K., Marosevic D., Bannister S.A., Bormane A., Donaghy M., Sing A. (2020). Longitudinal study of prevalence and spatio-temporal distribution of *Borrelia burgdorferi* sensu lato in ticks from three defined habitats in Latvia, 1999–2010. Environ. Microbiol..

[54] De Michelis S., Sewell H.S., Collares-Pereira M., Santos-Reis M., Schouls L.M., Benes V., Holmes E.C., Kurtenbach K. (2000). Genetic diversity of *Borrelia burgdorferi* sensu lato in ticks from mainland Portugal. J. Clin. Microbiol..

[55] Zeidner N.S., Schneider B.S., Nuncio M.S., Gern L., Piesman J. (2002). Coinoculation of *Borrelia* spp. with tick salivary gland lysate enhances spirochete load in mice and is tick species-specific. J. Parasitol..

[56] Collares-Pereira M., Couceiro S., Franca I., Kurtenbach K., Schafer S.M., Vitorino L., Goncalves L., Baptista S., Vieira M.L., Cunha C. (2004). First isolation of *Borrelia lusitaniae* from a human patient. J. Clin. Microbiol..

[57] Da Franca I., Santos L., Mesquita T., Collares-Pereira M., Baptista S., Vieira L., Viana I., Vale E., Prates C. (2005). Lyme borreliosis in Portugal caused by *Borrelia lusitaniae*? Clinical report on the first patient with a positive skin isolate. Wiener Klinische Wochenschrift.

[58] Lopes de Carvalho I.L., Fonseca J.E., Marques J.G., Ullmann A., Hojgaard A., Zeidner N., Nuncio M.S. (2008). Vasculitis-like syndrome associated with *Borrelia lusitaniae* infection. Clin. Rheumatol..

[59] Grego E., Bertolotti L., Peletto S., Amore G., Tomassone L., Mannelli A. (2007). *Borrelia lusitaniae OspA* gene heterogeneity in Mediterranean basin area. J. Mol. Evol..

[60] Estrada-Peña A., Nava S., Petney T. (2014). Description of all the stages of *Ixodes inopinatus* n. sp. (Acari: *Ixodidae*). Ticks Tick Borne Dis..

[61] Noureddine R., Chauvin A., Plantard O. (2010). Lack of genetic structure among Eurasian populations of the tick *Ixodes ricinus* contrasts with marked divergence from north-African populations. Int. J. Parasitol..

[62] Falco R.C., Fish D. (1992). A comparison of methods for sampling the deer tick, *Ixodes dammini*, in a Lyme disease endemic area. Exp. Appl. Acarol..

[63] Rollins R.E., Mouchet A., Margos G., Fingerle V., Becker N.S., Dingemanse N.J. (2021). Repeatable differences in exploratory behaviour predict tick infestation probability in wild great tits. Behav. Ecol. Sociobiol..

[64] Estrada-Peña A., Bouattour A., Camicas J.L., Walker A.R. (2004). Ticks of Domestic Animals in the Mediterranean Region—A Guide to Identification of Species.

[65] Guy E.C., Stanek G. (1991). Detection of *Borrelia burgdorferi* in patients with Lyme disease by the polymerase chain reaction. J. Clin. Pathol..

[66] Derdakova M., Beati L., Pet’ko B., Stanko M., Fish D. (2003). Genetic variability within *Borrelia burgdorferi* sensu lato genospecies established by PCR-single-strand conformation polymorphism analysis of the *rrfA*-*rrlB* intergenic spacer in *Ixodes ricinus* ticks from the Czech Republic. Appl. Environ. Microbiol..

[67] Rijpkema S.G., Molkenboer M.J., Schouls L.M., Jongejan F., Schellekens J.F. (1995). Simultaneous detection and genotyping of three genomic groups of *Borrelia burgdorferi* sensu lato in Dutch *Ixodes ricinus* ticks by characterization of the amplified intergenic spacer region between 5S and 23S rRNA genes. J. Clin. Microbiol..

[68] Johnson B.J., Happ C.M., Mayer L.W., Piesman J. (1992). Detection of *Borrelia burgdorferi* in ticks by species-specific amplification of the flagellin gene. Am. J. Trop. Med. Hyg..

[69] Hidri N., Barraud O., de Martino S., Garnier F., Paraf F., Martin C., Sekkal S., Laskar M., Jaulhac B., Ploy M.C. (2012). Lyme endocarditis. Clin. Microbiol. Infect..

[70] Margos G., Gatewood A.G., Aanensen D.M., Hanincova K., Terekhova D., Vollmer S.A., Cornet M., Piesman J., Donaghy M., Bormane A. (2008). MLST of housekeeping genes captures geographic population structure and suggests a European origin of *Borrelia burgdorferi*. Proc. Natl. Acad. Sci. USA.

[71] Margos G., Vollmer S.A., Cornet M., Garnier M., Fingerle V., Wilske B., Bormane A., Vitorino L., Collares-Pereira M., Drancourt M. (2009). A new *Borrelia* species defined by Multilocus Sequence Analysis of Housekeeping Genes. Appl. Environ. Microbiol..

[72] Mangold A.J., Bargues M.D., Mas-Coma S. (1998). Mitochondrial 16S rDNA sequences and phylogenetic relationships of species of *Rhipicephalus* and other tick genera among Metastriata (Acari: *Ixodidae*). Parasitol. Res..

[73] Huson D.H., Bryant D. (2006). Application of phylogenetic networks in evolutionary studies. Mol. Biol. Evol..

[74] Croucher N.J., Page A.J., Connor T.R., Delaney A.J., Keane J.A., Bentley S.D., Parkhill J., Harris S.R. (2015). Rapid phylogenetic analysis of large samples of recombinant bacterial whole genome sequences using Gubbins. Nucleic Acids Res..

[75] Bruen T.C., Philippe H., Bryant D. (2006). A simple and robust statistical test for detecting the presence of recombination. Genetics.

[76] Grana-Miraglia L., Evans B.A., Lopez-Jacome L.E., Hernandez-Duran M., Colin-Castro C.A., Volkow-Fernandez P., Cevallos M.A., Franco-Cendejas R., Castillo-Ramirez S. (2020). Origin of OXA-23 Variant OXA-239 from a Recently Emerged Lineage of *Acinetobacter baumannii* International Clone V. mSphere.

[77] Cheng L., Connor T.R., Sirén J., Aanensen D.M., Corander J. (2013). Hierarchical and spatially explicit clustering of DNA sequences with BAPS software. Mol. Biol. Evol..

[78] Stecher G., Tamura K., Kumar S. (2020). Molecular Evolutionary Genetics Analysis (MEGA) for macOS. Mol. Biol. Evol..

[79] James G., Witten D., Hastie T., Tibshirani R. (2013). An Introduction to Statistical Learning.

[80] Gower J.C. (1971). A general coefficient of similarity and some of its properties. Biometrics.

[81] R Core Team (2014). R: A Language and Environment for Statistical Computing.

[82] Galili T. (2015). Dendextend: An R package for visualizing, adjusting and comparing trees of hierarchical clustering. Bioinformatics.

[83] Francisco A.P., Vaz C., Monteiro P.T., Melo-Cristino J., Ramirez M., Carrico J.A. (2012). PHYLOViZ: Phylogenetic inference and data visualization for sequence based typing methods. BMC Bioinform..

[84] Francisco A.P., Bugalho M., Ramirez M., Carrico J.A. (2009). Global optimal eBURST analysis of multilocus typing data using a graphic matroid approach. BMC Bioinform..

[85] Darriba D., Taboada G.L., Doallo R., Posada D. (2012). jModelTest 2: More models, new heuristics and parallel computing. Nat. Methods.

[86] Guindon S., Gascuel O. (2003). A simple, fast, and accurate algorithm to estimate large phylogenies by maximum likelihood. Syst. Biol..

[87] Pérez-Eid C. (2007). Les Tiques: Identification, Biologie, Importance Médicale et Veterinaire.

[88] Altschul S.F., Gish W., Miller W., Myers E.W., Lipman D.J. (1990). Basic local alignment search tool. J. Mol. Biol..

[89] Jacquot M., Gonnet M., Ferquel E., Abrial D., Claude A., Gasqui P., Choumet V., Charras-Garrido M., Garnier M., Faure B. (2014). Comparative population genomics of the *Borrelia burgdorferi* species complex reveals high degree of genetic isolation among species and underscores benefits and constraints to studying intra-specific epidemiological processes. PLoS ONE.

[90] Shapiro B.J., Friedman J., Cordero O.X., Preheim S.P., Timberlake S.C., Szabo G., Polz M.F., Alm E.J. (2012). Population genomics of early events in the ecological differentiation of bacteria. Science.

[91] Shapiro B.J. (2014). Signatures of natural selection and ecological differentiation in microbial genomes. Adv. Exp. Med. Biol..

[92] Frey W., Lösch R. (2010). Geobotanik—Pflanze und Vegetation in Raum und Zeit.

[93] Poli P., Lenoir J., Plantard O., Ehrmann S., Roed K.H., Leinaas H.P., Panning M., Guiller A. (2020). Strong genetic structure among populations of the tick *Ixodes ricinus* across its range. Ticks Tick Borne Dis..

[94] Carranza S., Harris D.J., Arnold E.N., Batista V., Gonzalez de la Vega J.P. (2006). Phylogeography of the lacertid lizard, *Psammodromus algirus*, in Iberia and across the Strait of Gibraltar. J. Biogeogr..

[95] Verdú Ricoy J., Carranza S., Salvador A., Busack S., Díaz J. (2010). Phylogeography of *Psammodromus algirus* (*Lacertidae*) revisited: Systematic implications. Amphib. Reptil..

[96] Younsi H., Sarih M., Jouda F., Godfroid E., Gern L., Bouattour A., Baranton G., Postic D. (2005). Characterization of *Borrelia lusitaniae* isolates collected in Tunisia and Morocco. J. Clin. Microbiol..

