# Supplementary material for: The Population Structure of Borrelia lusitaniae Is Reflected by a Population Division of Its Ixodes Vector
Source: Microorganisms. 2021 Apr 27;9(5):933. doi: 10.3390/microorganisms9050933 (PMC8145215; doi:10.3390/microorganisms9050933)
Supplement: Supplementary file 1 [file microorganisms-09-00933-s001.zip › Norte et al_Supplementary Information_Methods_Figures_check.pdf]

Supplementary Information

Norte et al. 2021 Supplementary methods and results

Sample collection in Portugal.

In 2003 and 2013 ticks were collected in Tapada de Mafra which is an enclosed game area with controlled hunting populations of fallow deer (*Cervus dama*), red deer (*Cervus elaphus*) and wildboar (*Sus scrofa*). Its mixed forest comprises cork oaks (*Quercus suber*), Portuguese oaks (*Quercus faginea*), maritime pines (*Pinus pinaster*), poplars (*Populus sp.*), plane trees (*Platanus* sp.), ash (*Fraxinus* sp.) and wild olive trees (*Olea europaea* var. *sylvestris*). In the herbaceous stratum *Brachypodium phoenicoides* and the common bracken (*Pterydium aquilinum*) are found abundantly. One tick was collected in Madeira Archipelago, in Calheta, in 2009. Madeira archipelago is located in the north Atlantic Ocean about 1000 km from the European coast and 800 km west of Africa. Climatic conditions in this archipelago are ideal for *I. ricinus* ticks due to high humidity and mild temperatures. Parque Natural Peneda-Gerês (PNPG) is located on the northern border of Portugal and presents also adequate climatic conditions for *I. ricinus* populations. Here a tick was collected at Pedra Bela in 2009. The tick collected in Santiago do Cacém in 2009 was collected in the largest safari park (approximately 90 ha) in Portugal which harbours more than 40 species of exotic animals, including ungulates that are important tick reproduction hosts.

PCR conditions.

***Ixodes* genes.** TROSPA gene amplification was performed according to Noureddine et al. 2011. For samples processed in Slovakia, the PCR reaction mixture contained 2.5 µL of 10X PCR buffer, 1 µL of 25mM MgCl2, 0.125 µL of polymerase (Qiagen, Hilden, Germany), 0.5 µL of both primers (10 µM), 0.5 µL of 10mM dNTP (Thermofisher, Dreieich, Germany), 14.875 µL of nuclease-free water (Promega, Madison, WI, USA) and 5 µL of DNA as a template.

PCR for 16S rRNA was performed according to Mangold et al. 1998.

***Borrelia* loci.** For samples from Slovakia, Serbia, Urkaine, Latvia, Austria forward primer IGSA (5´-CGACCTTCTTCGCCTTAAAGC-3´) and reverse primer IGSB (5´-AGCTCTTATTCGCTGATGGTA-3´) primers were used for initial screening. PCR amplifications were performed in a total reaction mixture of 25 µL. The PCR reaction mixture per each sample contained of 2.5 µL of 10X PCR buffer, 1 µL of 25mM MgCl2, 0.125 µL of polymerase (Qiagen, Hilden, Germany), 0.5 µL of both primers (10 µM), 0.5 µL of 10mM dNTP (Thermofisher, Dreieich, Germany), 14.875 µL of nuclease-free water (Promega, Madison, WI, USA) and 5 µL of DNA as template. Touch-down PCR program consisted of these steps: Initial denaturation at 95 °C for 5 min, followed by 5 cycles of denaturation at 94 °C for 15 s, annealing at 61 °C (- 0.2 °C per cycle) for 25 s and elongation at 72 °C for 30 s. It was followed by 5 cycles of denaturation at 94 °C for 15 s, annealing at 60 °C (- 0.4 °C per cycle) for 25 s and elongation at 72 °C for 30 s. Then followed 30 cycles of denaturation (94 °C, 15 s), annealing (58 °C, 25 s) and elongation (72 °C, 30 s). The program was terminated by elongation at 72 °C for 5 min. The PCR products were electrophoresed on 1.5 % agarose gel. Than it was stained with GoodView (Ecoli, Bratislava, Slovakia) and visualized on UV transiluminator Vilber-Lourmant (Sigma-Aldrich, St. Louis, MO, USA).

For MLST PCR, HotStarTaq Plus DNA Polymerase Kit (Qiagen, Hilden, Germany) was used. The PCR reaction mixture per each sample for the first run contained: 2.5 µL buffer (10X), 1 µL MgCl2 (25mM), 1 µL 10 µM dNTP (Thermofisher, Dreieich, Germany), 0.15 µL HotStart Taq polymerase, 1 µL (10 µM) outer forward primer, 1 µL outer reverse primer, 15.85 µL nuclease-free water (Promega, Madison, WI, USA) and 2.5 µL DNA sample. PCR amplification was performed in a total reaction mixture of 25 µL. For the second round of PCR amplification (nested/seminested PCR), the reaction mixture per each sample contained of: 3 µL buffer (10X), 1.5 µL MgCl2 (25mM), 1.5 µL dNTP (10 µM), 0.27 µL HotStart Taq polymerase, 1.5 µL (10 µM) outer forward primer, 1.5 µL outer reverse primer, 17.73 µL nuclease-free water and 3 µL PCR product of the first run. PCR amplification was performed in a total reaction mixture of 30 µL. PCR amplifications were carried out on thermocycler BIO-RAD T100 Thermal Cycler (BIO-RAD, California, USA).

Prevalence of Borrelia. There are no prevalence data for Portugal and Croatia. In Slovakia, out of 150 *I. ricinus* ticks collected on Martinské hole, *B. burgdorferi* s.l. was detected in 22 (14.7 %) out of 150 ticks. *B. lusitaniae* was a dominant genospecies (10/22, 14.7 %), followed by *B. afzelii* (9/22, 40.9 %), *B. burgdorferi* s.s. (2/22, 9.1 %) and *B. garinii* (1/22, 4.5 %). In Ukraine spirochetes from the *B. burgdorferi* s.l. complex were found in 133 (25.8 %) of 515 examined ticks in Kyiv. Seven genospecies were identified: *B. afzelii* (117/133; 87 %), *B. garinii* (4/133; 3 %), *B. valaisiana* (2/133; 1.50 %), *B. bavariensis* (2/133; 1.50 %), *B. spielmanii* (2/133; 1.50 %), *B. burgdorferi* s.s. (1/133; 0.75 %) and *B. lusitaniae* (1/133; 0.75 %).

Supplementary Tables (see additional Excel file)

Table S1 *Ixodes* tick samples included in this study including information on processing of samples

Table S2 Borrelia samples included in this study, sequence type (ST) number (if available), allele numbers of the seven genes included for analyses and details of sample processing

Table S3 Detailed results of Hierarchical BAPS analyses at levels 1 and 2

Supplementary Figures (Fig S1 – S4)

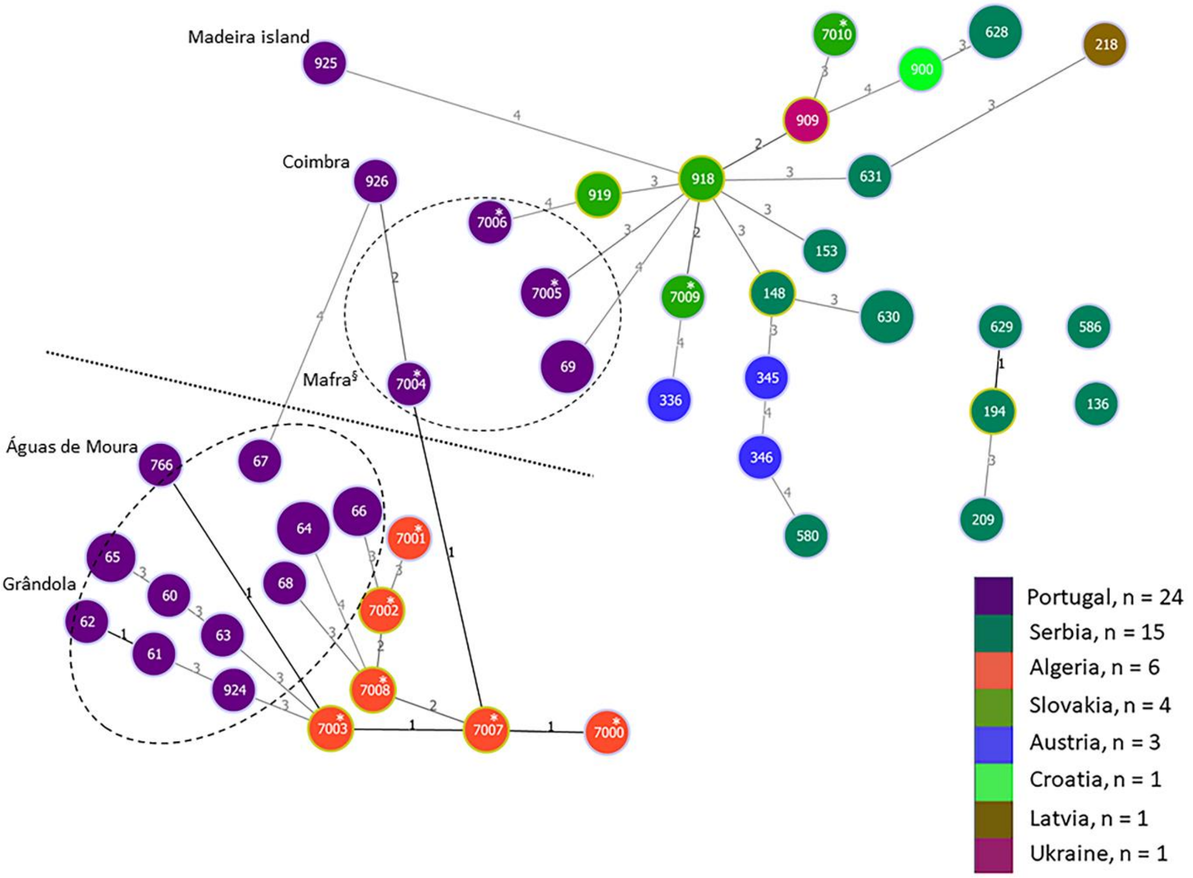

Figure S1 Phyloviz diagram [83] of *B. lusitaniae* samples based on allele profile for seven MLST genes. Links between samples were generated using higher level edges (here level 4 which is based on the inter-relationship of four alleles). Samples are color coded according to country of origin. For samples from Portugal, the region is indicated. The link color of edges are as follows: Link colors for goeBURST results: Black - Link drawn without recourse to tiebreak rules; Blue - Link drawn using tiebreak rule 1 (number of SLVs); Gray - Links drawn at DLV (darker gray) or TLV and higher level (lighter/dashed gray) if the groups are constructed at DLV/TLV level. * indicate STs derived from 7 genes only with no correspondence to MLST database ST numbers. § includes one isolate from a human. Dotted line separates samples from the north and south of river Tagus in Portugal.

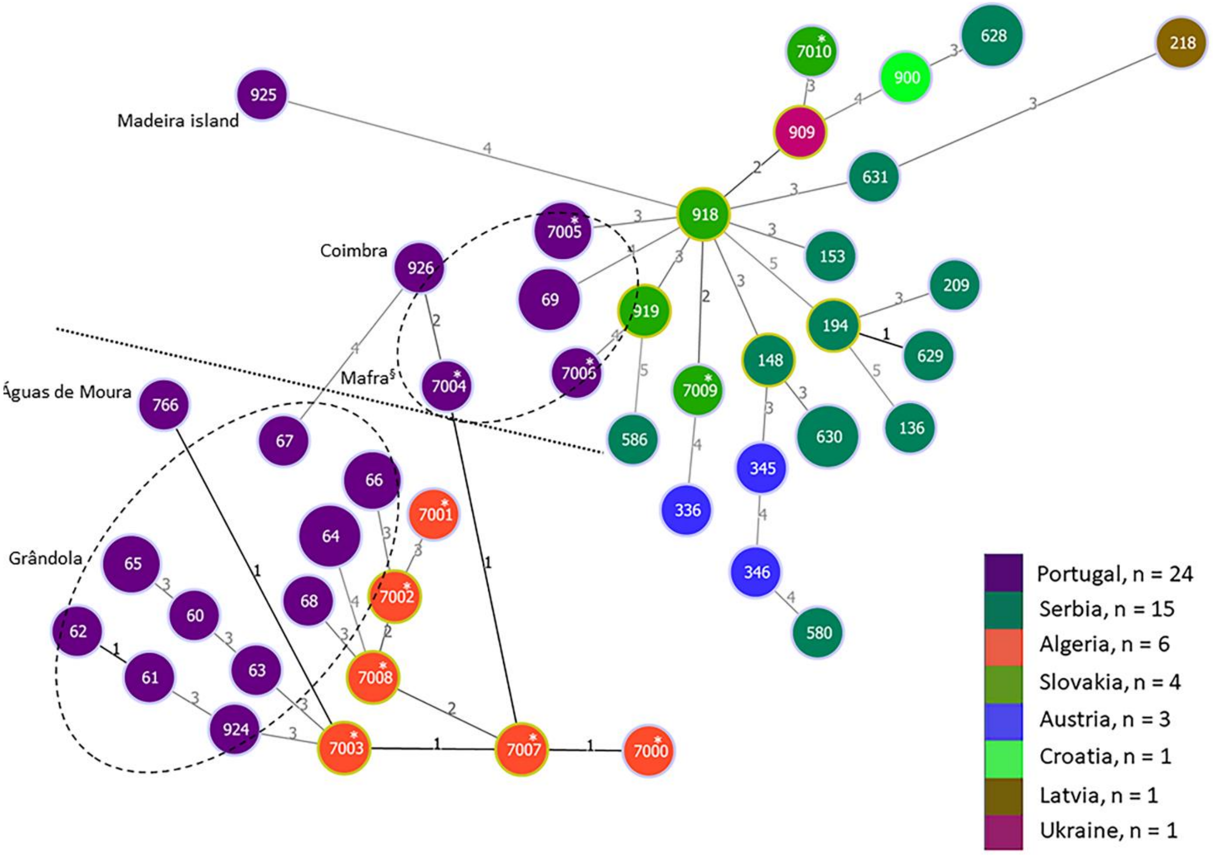

Figure S2 Phyloviz diagram [83] of *B. lusitaniae* samples based on allele profile for seven MLST genes. Links between samples were generated using higher level edges (here level 5 which is based on the inter-relationship of five alleles). Samples are color coded according to country of origin. For samples from Portugal, the region is indicated. The link color of edges are as follows: Link colors for goeBURST results: Black - Link drawn without recourse to tiebreak rules; Blue - Link drawn using tiebreak rule 1 (number of SLVs); Gray - Links drawn at DLV (darker gray) or TLV and higher level (lighter/dashed gray) if the groups are constructed at DLV/TLV level. * indicate STs derived from seven genes only with no correspondence to MLST database ST numbers. § includes one isolate from a human. Dotted line separates samples from the north and south of river Tagus in Portugal.

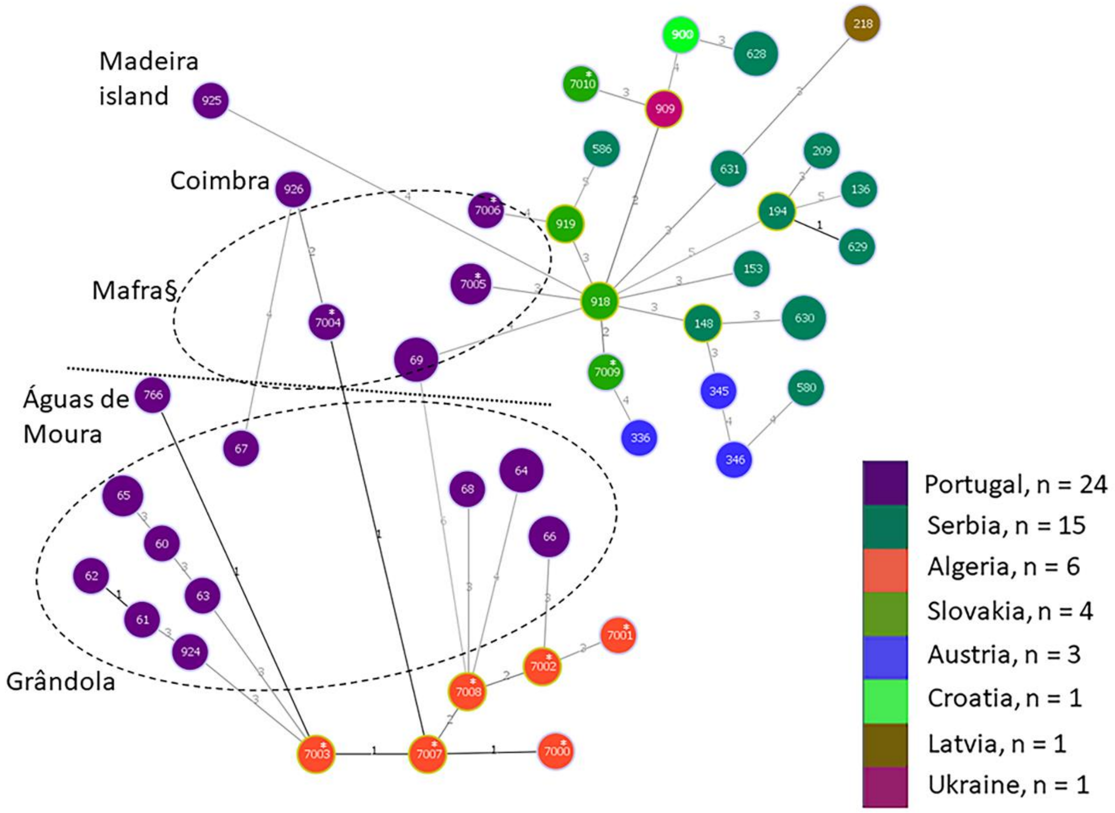

Figure S3: Phyloviz diagram [83] of *B. lusitaniae* samples based on allele profile for seven MLST genes. A full MST was created and level 6 setting chosen. Samples are color coded according to country of origin. For samples from Portugal, the region is indicated. Numbers with 700X indicate STs derived from 7 genes only with no correspondence to MLST database ST numbers.

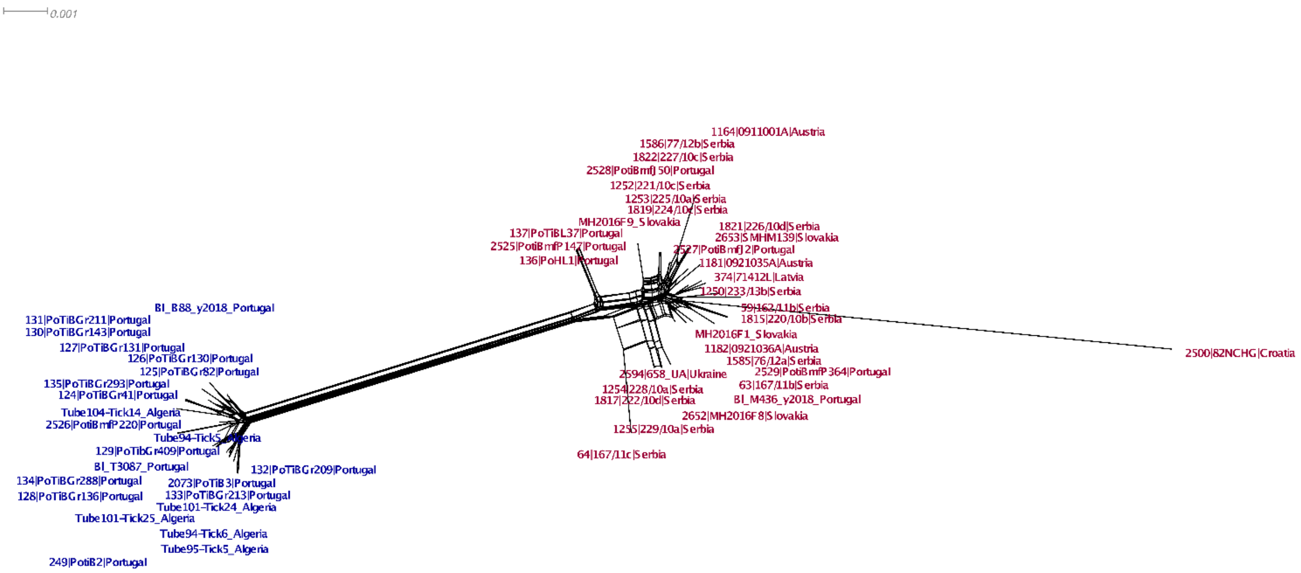

Figure S4. NeighborNet of the MLST alignment generated in Splitstree4 [73]. The net was yielded considering uncorrected P distance and equal angle display. Blue color indicates the major group that includes the North African and some Portuguese isolates, whereas red color shows the other major group containing most of the European isolates. The different color labels indicate the clusters found at the second level of clustering of the hierarchical population structure analysis using hierBAPS. The scale bar shows the number of substitutions per site.
